# Supplementary material for: Desert dust exerts twice the longwave radiative heating estimated by climate models
Source: Nat Commun. 2026 Apr 28;17:3191. doi: 10.1038/s41467-026-70952-9 (PMC13125314; doi:10.1038/s41467-026-70952-9)
Supplement: Supplementary file 1 — Supplementary Information [file 41467_2026_70952_MOESM1_ESM.pdf]

Supplementary Information for "Desert dust exerts twice the longwave radiative heating estimated by climate models"

Jasper F. Kok[1,*], Ashok. K. Gupta[1,2], Amato T. Evan[3], Carlos Pérez García-Pando[4,5], Longlei Li[6], Adeyemi A. Adebiyi[7], Samuel Albani[8], Yves Balkanski[9], Ramiro Checa-Garcia[10], Peter R. Colarco[11], Douglas S. Hamilton[12], Yue Huang[1], Akinori Ito[13], Martina Klose[14], Natalie M. Mahowald[6], Ron L. Miller[15], Vincenzo Obiso[4], Adriana Rocha Lima[16], Jessica Wan[6,17]

[1]Department of Atmospheric and Oceanic Sciences, University of California, Los Angeles, CA 90095, USA

[2]Department of Earth and Environmental Sciences, Vanderbilt University, Nashville, TN 37240, USA

[3]Scripps Institution of Oceanography, University of California, San Diego, CA 92093, USA

[4]Barcelona Supercomputing Center (BSC), 08034 Barcelona, Spain

[5]ICREA, Catalan Institution for Research and Advanced Studies, 08010 Barcelona, Spain

[6]Department of Earth and Atmospheric Sciences, Cornell University, Ithaca, NY, 14850, USA.

[7]Department of Life and Environmental Sciences, University of California, Merced, CA, 95344, USA

[8]Department of Environmental and Earth Sciences, University of Milano-Bicocca, Milano, Italy

[9]Laboratoire des Sciences du Climat et de l'Environnement, CEA-CNRS-UVSQ-UPSaclay, Gif-sur-Yvette, France

[10]Royal Netherlands Meteorological Institute, De Bilt, the Netherlands

[11]Atmospheric Chemistry and Dynamics Laboratory, NASA Goddard Space Flight Center, Greenbelt, MD 20771, USA

[12]Marine, Earth, and Atmospheric Science, North Carolina State University, Raleigh, NC, 27606, USA

[13]Yokohama Institute for Earth Sciences, JAMSTEC, Yokohama, Kanagawa 236-0001, Japan

[14]Institute of Meteorology and Climate Research Troposphere Research, Karlsruhe Institute of Technology (KIT), Karlsruhe, Germany

[15]NASA Goddard Institute for Space Studies, New York NY10025 USA

[16]Department of Physics, University of Maryland, Baltimore County, 1000 Hilltop Circle, Baltimore, MD, 21250, USA

[17]Climate Systems Engineering initiative, University of Chicago, Chicago, IL 60637, USA

[*]Corresponding author: jfkok@ucla.edu

Supplementary Methods

Below, we describe the DustCOMM dust climatology data set, the various effective emission temperatures used in the analytical model, how the radiative transfer calculations of the atmospheric absorptivity were performed, how the bootstrap procedure was performed, and the treatment of longwave interactions in our ensemble of six global model simulations that were also used in obtaining the DustCOMM data set. We end with a discussion of the limitations of our methodology.

DustCOMM dust climatology data set. One of the main data sets used in the analytical model is the Dust Constraints from joint Observational-Modelling-Experimental analysis (DustCOMM) data set. Details on this data set can be found in various recent publications[1-4] and we provide a brief overview here. DustCOMM is a climatology of the global dust cycle obtained for the years 2004-2008. It provides constraints on the main properties of the global dust cycle, including dust concentration, dust aerosol optical depth, and dust deposition fluxes. All these variables are resolved by season, particle size (up to a diameter of 20 μm), and the emitting major source region.

The DustCOMM data set was produced using inverse modeling, which integrated an ensemble of simulations from six global models (listed in table 1 in Kok et al.[3] and also in Supplementary Table 3) with observational constraints on the dust size distribution[1], extinction efficiency[5], and regional dust aerosol optical depth near dust source regions[2,6]. The DustCOMM data include uncertainties, which were obtained through a bootstrap procedure[7] that propagated uncertainty from the spread in the model simulations, uncertainties in observed dust microphysical properties, and uncertainties in the regional DAOD. Comparisons against dust surface concentration and deposition flux measurements indicated that DustCOMM is in substantially better agreement with these independent measurements than current model simulations[3].

For this paper, we used three products from the DustCOMM data set (Supplementary Fig. 2). First, we used the dust concentration, resolved by location, particle size, and season, which we combined with data sets of the dust LW refractive index (Supplementary Table 6) to calculate the LW dust aerosol optical depth (Supplementary Fig. 11) and ultimately the dust clear-sky LW DRE. Second, we used the SW DAOD, which we combined with the clear-sky LW DRE to calculate the clear-sky LW DREE, which we then compared against observations. Note that some of the model simulations used in DustCOMM assumed that the clear-sky SW DAOD equals the all-sky SW DAOD, which could cause errors, although studies indicate that such a systematic difference is small for dusty regions[6,8]. And finally, we used the ratio of the clear-sky to the all-sky LW DRE from the six models used in the DustCOMM data set, which we combined with the clear-sky LW DRE calculated by the data-driven analytical model to obtain the all-sky LW DRE.

Effective emission temperatures. We define the effective emission temperature as the temperature that a blackbody would need to have to emit the same radiative flux in the atmospheric window (taken as 8-14 μm). The effective emission temperature T_{d} (Supplementary Fig. 12e) of dust in an atmospheric column is then

$$T_{\mathrm{d}} = \left[\frac{1}{f_{\mathrm{aw}}(T_{\mathrm{d}})}\frac{\sum_{i=1}^{N_{\mathrm{i}}}\bar{\tau}_{\mathrm{d},i}f_{\mathrm{aw}}(T_i)T_i^4}{\sum_{i=1}^{N_i}\bar{\tau}_{\mathrm{d},i}}\right]^{\frac{1}{4}}, \tag{1}$$

where f_{aw} (Supplementary Fig. 13) is the fraction of the emitted radiative flux with wavelength in the atmospheric window, the index *i* sums over the $N_{\mathrm{i}} = 48$ pressure levels in the DustCOMM data set spanning from sea level to the top-of-atmosphere, and T_i is the atmospheric temperature at the center of each pressure level, which is supplied by MERRA-2 reanalysis data[9]. For simplicity, Supplementary Equation (1) assumes that the total column dust optical depth $\tau_d \ll 1$, such that a weighting function to calculate the fraction of radiation from each model level that transmits to the top of the dust layer is not needed; the impact of the assumption of $\tau_d \ll 1$ is discussed further below. In addition, $\bar{\tau}_{\mathrm{d},i}$ is the optical thickness (unitless) of dust in layer *i*, which is calculated as

$$\bar{\tau}_{\mathrm{d},i} = \sum_{b=1}^{N_b} l_{i,b}\,\bar{k}_{\mathrm{ext},b}, \tag{2}$$

where *b* sums over the N_{b} dust particle bins (Supplementary Table 4), which include the bins spanning until 20 μm diameter in the DustCOMM product[3] and three more bins that account for dust with diameter between 20 – 100 μm based on CESM simulations calibrated to in situ measurements of super coarse dust[10,11]. Furthermore, $l_{i,b}$ is the mass path (kg m^{-2}) of bin *b* in vertical model layer *i*, and $\bar{k}_{\mathrm{ext},b}$ is each bin's mass extinction efficiency (m^2 kg^{-1}) in the atmospheric window, which was obtained as described below and listed in Supplementary Table 4.

We similarly define the effective emission temperature of upwelling atmospheric radiation below the dust layer (T_{bel}) and of downwelling radiation above the dust layer (T_{abv}) as the temperature that a blackbody would need to have to produce the same radiative flux in the atmospheric window. That is,

$$T_{\mathrm{bel}} = \left[\frac{1}{f_{\mathrm{aw}}(T_{\mathrm{bel}})}\frac{\sum_{i=1}^{i_{\mathrm{d}}-1}\bar{\epsilon}_{\mathrm{atm},i}f_{\mathrm{aw}}(T_i)T_i^4}{\sum_{i=1}^{N_i}\bar{\epsilon}_{\mathrm{atm},i}}\right]^{\frac{1}{4}}, \text{ and} \tag{3}$$

$$T_{\mathrm{abv}} = \left[\frac{1}{f_{\mathrm{aw}}(T_{\mathrm{abv}})}\frac{\sum_{i_d+1}^{N_i}\bar{\epsilon}_{\mathrm{atm},i}f_{\mathrm{aw}}(T_i)T_i^4}{\sum_{i=1}^{N_i}\bar{\epsilon}_{\mathrm{atm},i}}\right]^{\frac{1}{4}}, \tag{4}$$

where $\bar{\epsilon}_{\mathrm{atm},i}$ is the absorptivity due to atmospheric constituents in model layer *i* below the dust layer's central model layer i_{d}, calculated using a radiative transfer model (see below); T_{bel} is typically one to ten degrees colder than T_{s}.

The effective emission temperature below the dust layer, $T_{\mathrm{s,eff}}$ (Supplementary Fig. 12c), depends primarily on the surface temperature T_{s} (Supplementary Fig. 12a) and surface emissivity ϵ_{s} (Supplementary Fig. 12b); $T_{\mathrm{s,eff}}$ - T_{s} can be up to ~5 °C in desert regions with relatively small ϵ_{s} (Supplementary Figs. 12b, 12d). The effective emission temperature is defined as

$$T_{\mathrm{s,eff}} = \left[\frac{(1-\bar{\epsilon}_{\mathrm{bel}})}{f_{\mathrm{aw}}(T_{\mathrm{s,eff}})}\left[\bar{\epsilon}_{\mathrm{s}}f_{\mathrm{aw}}(T_{\mathrm{s}})T_{\mathrm{s}}^4 + (1-\bar{\epsilon}_{\mathrm{s}})f_{\mathrm{aw}}(T_{\mathrm{atm}\downarrow})T_{\mathrm{atm}\downarrow}^4\right] + \frac{f_{\mathrm{aw}}(T_{\mathrm{bel}})}{f_{\mathrm{aw}}(T_{\mathrm{s,eff}})}\bar{\epsilon}_{\mathrm{bel}}T_{\mathrm{bel}}^4\right]^{\frac{1}{4}}, \tag{5}$$

where $T_{\mathrm{atm}\downarrow}$ is the effective emission temperature of downwelling radiation at the surface (generally, $T_{\mathrm{atm}\downarrow} < T_s$). The three terms in Supplementary Equation (5) respectively represent

the contributions of upwelling radiation emitted by the surface, of the downwelling atmospheric radiation scattered upward by the surface, and of the upwelling radiation emitted by the atmosphere between the surface and the dust layer.

Finally, the effective emission temperature of downwelling radiation at the surface equals

$$T_{\text{atm}\downarrow} = \left[\frac{1}{f_{\text{aw}}(T_{\text{atm}\downarrow})}\left[\bar{\epsilon}_{\text{bel}} f_{\text{aw}}(T_{\text{bel}}) T_{\text{bel}}^4 + (1-\bar{\epsilon}_{\text{bel}}-\bar{\epsilon}_{\text{d}})\bar{\epsilon}_{\text{abv}} f_{\text{aw}}(T_{\text{abv}}) T_{\text{abv}}^4 + (1-\bar{\epsilon}_{\text{bel}})\bar{\epsilon}_{\text{d}} f_{\text{aw}}(T_{\text{d}}) T_{\text{d}}^4 + (1-\bar{\epsilon}_{\text{bel}}) f_{\text{aw}}(T_{\text{s,eff}}) T_{\text{s,eff}}^4 R_d\right]\right]^{\frac{1}{4}}, \tag{6}$$

where the first two of the four terms inside the square brackets respectively represents the contributions from the atmosphere below and above the dust layer, the third term represent the contribution of emission from the dust layer, and the fourth term represents the (relatively small) contribution of upwelling radiation below the dust layer that is scattered down towards Earth's surface by the dust layer.

Dust optical properties. The optical properties in the LW spectrum are quite uncertain, in large part because of a scarcity of measurements. Correspondingly, values of the refractive index in the longwave spectrum used in different models and theoretical studies vary greatly, as summarized in Di Biagio et al.[12]. Considering this large divergence, we draw from six different LW refractive index data sets in common use in the literature[12-17]. For each of these data sets, we obtained the complex refractive index averaged over the atmospheric window (Supplementary Table 6). We then calculated the mass extinction efficiency, downscatter fraction, and single-scattering albedo for each bin b ($\bar{k}_{\text{ext},b}$, $\bar{\beta}_{\downarrow,b}$, and $\bar{\omega}_b$, respectively) as

$$\bar{k}_{\text{ext},b} = \frac{\int_{D_{b-}}^{D_{b+}} \frac{dN}{dD}\frac{\pi}{4}D^2\, \bar{Q}_{\text{ext}}(D)dD}{\int_{D_{b-}}^{D_{b+}} \frac{dN}{dD}\frac{\pi}{6}D^3 \rho_{\text{d}} dD}, \tag{7}$$

$$\bar{\beta}_{\downarrow,b} = \frac{\int_{D_{\text{min},b}}^{D_{b+}} \frac{dN}{dD}\frac{\pi}{4}D^2\, \bar{Q}_{\text{scat}}(D)\bar{\beta}_{\downarrow}(D)dD}{\int_{D_{b-}}^{D_{b+}} \frac{dN}{dD}\frac{\pi}{4}D^2\, \bar{Q}_{\text{scat}}(D)dD}, \tag{8}$$

$$\bar{\omega}_b = \frac{\int_{D_{b-}}^{D_{b+}} \frac{dN}{dD}\frac{\pi D^2}{4}\, \bar{Q}_{\text{ext}}(D)\bar{\omega}(D)dD}{\int_{D_{b-}}^{D_{b+}} \frac{dN}{dD}\frac{\pi}{4}D^2\, \bar{Q}_{\text{ext}}(D)dD}, \tag{9}$$

where D_{b-} and D_{b+} are respectively the lower and upper diameter limits of particle size bin k, $\rho_d = (2.5 \pm 0.2) \times 10^3$ kg m^{-3} is the globally representative density of dust aerosols[18-21], and $\frac{dN}{dD}$ is the observationally constrained globally averaged dust number size distribution obtained in Adebiyi and Kok[1]. Furthermore, $\bar{Q}_{\text{scat}}(D)$, $\bar{Q}_{\text{ext}}(D)$, $\bar{\beta}_{\downarrow}(D)$ and $\bar{\omega}(D)$ are respectively the size-resolved scattering efficiency, extinction efficiency, downscatter fraction, and single-scattering albedo. Note that the exact values of these optical properties for a given model bin vary for each bootstrap iteration (explained further below) as they depend on which of the complex refractive indices in Supplementary Table 6 was drawn in the bootstrap procedure as well on which realization of the sub-bin dust size distribution was drawn from the ensemble of possible global dust size distributions provided in Adebiyi and Kok[1]. As such, the bootstrap procedure

(described further below) propagates the uncertainty in the LW refractive index into our calculation of the LW DRE.

Supplementary Table 4 reports the mean values and standard deviation of the mass extinction efficiency ($\bar{k}_{\mathrm{ext},b}$), downscatter fraction ($\bar{\beta}_{\downarrow,b}$), and single-scattering albedo ($\bar{\varpi}_b$) for each bin. Variability in these optical properties for a given particle bin are due to variability in the sub-bin dust size distribution [see Supplementary Equations. (7)-(9)], and in the dust complex refractive index in the atmospheric window, based on a random drawing of one of six data sets (see Supplementary Table 6).

Note that the optical properties calculated by Supplementary Equations. (7)-(9) neglect the effect of dust asphericity. This is necessary because there are no optical properties available for aspherical dust for the broad range of complex refractive indices used in this study (Supplementary Table 5), necessitating the use of Mie theory and therefore the assumption of spherical dust particles. The main effect of neglecting dust asphericity is an underestimation of the mass extinction efficiency by ~40% in both the SW[5] and LW spectra[22]. In other words, asphericity has a minimal effect on the ratio of the LW to the SW DAOD (Supplementary Fig. 14), which co-determines the LW radiative effects (Equation 13) because the main constraint on the size of the global dust cycle is the SW DAOD[3]. For consistency, we therefore also neglected the effect of asphericity in the SW spectrum. That is, we obtained the DustCOMM data as described in Kok et al.[3], except that we used optical properties in the SW spectrum obtained from Mie theory with the SW complex refractive index used in Kok et al.[5]. Because dust abundance in the DustCOMM climatology is constrained by the SW DAOD[6], this results in an enhancement of the global dust mass loading by ~40%, which almost exactly counteracts the reduction in LW DAOD that would be caused by neglecting the asphericity on LW optical properties only. As such, the effect of neglecting asphericity on the results reported in this paper is expected to induce an error that is small compared to other errors in the analysis (see discussion of limitations below).

Radiative transfer model simulations of atmospheric absorptivity. To assess the spatial and temporal variations of average atmospheric absorptivity ($\bar{\epsilon}_{\mathrm{atm}}$ and $\bar{\epsilon}_{\mathrm{abv}}$) and downwelling atmospheric temperature ($T_{\mathrm{atm}\downarrow}$) for clear-sky conditions within the 8–14 µm wavelength range, we employed the LibRadtran radiative transfer model[23,24]. Atmospheric profiles of water vapor, ozone, pressure, temperature, and air density were obtained from MERRA-2 reanalysis data[9] and were interpolated onto the grid used for DustCOMM[3]. Additionally, we used seasonal mean climatological values for trace gases, including CO_2, O_2, CH_4, and NO_2, from the Air Force Geophysics Laboratory (AFGL)[25] to represent background concentrations appropriate for radiative transfer simulations[23]. These three-dimensional atmospheric profiles of greenhouse gas concentrations, temperature, and pressure were then utilized as inputs for LibRadtran[24].

The model was configured to simulate thermal radiation specifically within the 8–14 µm atmospheric window, where water vapor, ozone, and CO_2 are the predominant absorbers. For the simulations, the six streams DISORT solver was used to perform accurate multi-layer radiative transfer calculations, while the REPTRAN molecular absorption parameterization was applied to achieve detailed spectral resolution[26,27]. These calculations were conducted over the period from 2004 to 2008, for which the DustCOMM dust climatology was obtained.

Bootstrap procedure to propagate uncertainties. In order to propagate the uncertainties in the inputs to our data-driven analytical model (orange boxes in Supplementary Fig. S2), we used the following bootstrap procedure[28,29]:

1. We randomly choose one the many realizations of the global dust cycle in the DustCOMM data set[3]. These realizations were themselves obtained from a bootstrap procedure that propagated uncertainties due to inputs to the DustCOMM data set, including on DAOD in 15 dusty regions[2,6], the globally averaged atmospheric dust particle size distribution in the atmosphere[1], and the spread between the six global model simulations used in the DustCOMM inversion method.
2. We randomly drew a complex refractive index representative of the atmospheric window from the six available data sets (Supplementary Table 6) and used it to calculate the LW optical properties ($\overline{\omega}_b$, $\bar{\beta}_{\downarrow,b}$, and $\bar{k}_{\text{ext},b}$).
3. Using this realization of the global dust cycle and the LW optical properties, we calculated the seasonally and spatially resolved LW clear-sky DRE at TOA, using the data-driven analytical model.
4. We calculated the root mean-squared error (RMSE) of the simulated LW clear-sky DREE relative to the compilation of LW DREE observations (Supplementary Table 5). If the RMSE was larger than $\text{RMSE}_{\text{max}} = 4\ \text{Wm}^{-2}$ (see Methods) then the bootstrap iteration was reinitialized at step 1. This resulted in the elimination of ~55% of the simulations, retaining the other ~45% (Supplementary Fig. 3).
5. We randomly drew one of the six global model simulations used in the DustCOMM data set and obtained $\eta(s, \theta, \phi)$, the spatially and seasonally resolved ratio of the simulated LW clear-sky DRE and LW all-sky DRE. We then used this to calculate the seasonally and spatially resolved LW all-sky DRE (see Equation 12 in Methods).

We repeated the above steps 1,000 times, yielding a large number of realizations that represent the probability distributions of the LW clear-sky and all-sky DRE, with the spread in these probability distributions representing the uncertainty due to the propagation of the various uncertainties in the input data to our procedure. We report the median and 90% confidence interval of these results in the main text. Because our procedure cannot propagate systematic errors due to limitations of our method, which are discussed in more detail below, the errors should be interpreted as a lower bound.

Treatment of LW interactions in the six global aerosol models. Below, we describe the treatment of LW interactions of each of the six models used in the DustCOMM inversion method, namely CESM/CAM4, IMPACT, GISS Model E2.1, GEOS/GOCART, MONARCH, and LMDZOR-INCA. Other details of these simulations can be found in the Supplement to Kok et al.[3]. The seasonal LW DREE simulated by each model is shown in Supplementary Fig. 15, with the comparison against the compilation of LW DREE observations (Supplementary Table 5) shown in Fig. 2b.

CESM. We use simulations with the Community Atmosphere Model version 4 (CAM4) within the Community Earth System Model version 1 (CESM1), which includes active atmosphere, land, and sea ice components, alongside a data ocean and slab glacier forced by MERRA2 meteorology. CAM4 utilizes the Bulk Aerosol Model (BAM) parameterization for dust size distribution[30], where emission fluxes are partitioned into four size bins (diameters: 0.1-1.0, 1.0-2.5, 2.5-5.0, 5.0-10 μm)[31]. Dust emissions in these bins follow the brittle fragmentation theory[32].

CAM4's longwave radiation scheme uses an absorptivity/emissivity formulation[33] that neglects aerosol scattering. A seven-band broadband approach is used, which accounts for water vapor window regions[34]. The longwave optical properties of dust in CAM4 are inherited from CAM3[31,35] and are based on Maxwell-Garnett mixing of 47.6% quartz, 25% illite, 25% montmorillonite, 2% calcite, and 0.4% hematite by volume. The prescribed dust density and hygroscopicity are 2500 kg m^{-3} and 0.14, respectively. To account for longwave aerosol scattering, the longwave dust direct radiative effect at the top of the atmosphere is scaled up by the maximum reported error of approximately 50%[36].

The cloud parameterization in CAM 4.0 diagnoses cloud fraction based on relative humidity, atmospheric stability, water vapor, and convective mass fluxes[37]. It categorizes clouds into three types: low-level marine stratocumulus, convective clouds, and layered clouds. Marine stratocumulus clouds are determined using empirical relationships involving potential temperature differences[38], while convective clouds are linked to updraft mass fluxes from deep and shallow cumulus schemes[39]. Layered clouds form when relative humidity exceeds a pressure-dependent threshold, with adjustments made to account for land-surface variability and cold climates to avoid unrealistic cloud decks[40]. The total cloud fraction combines these cloud types under a maximum overlap assumption, ensuring consistency between cloud fraction, condensate, and relative humidity.

IMPACT. This study used the Integrated Massively Parallel Atmospheric Chemical Transport (IMPACT) model to calculate the concentration of mineral dust aerosols in 4 size bins (diameters: 0.1–1.26, 1.26–2.5, 2.5–5, and 5–20 µm) (Ito et al.[41] and references therein), as in Kok et al.[3]. Emitted dust particles were distributed among these four bins following brittle fragmentation theory[32]. We used an off-line radiative transfer model to calculate the optical depth of mineral dust particles per layer and their resulting radiative effects (Ito et al., 2018 and references therein). The radiative parameterizations include effects of clouds based on the National Center for Atmospheric Research (NCAR) Community Atmosphere Model 3[43]. In this study, the off-line radiative transfer model estimated LW radiative effect based on CAM4 and thus scattering of longwave radiation by dust was neglected (Albani et al.[44] and references therein). In the off-line radiative transfer model, the mineral dust particles were treated as externally mixed in each size bin, and thus the water uptake by dust particles was neglected. The aerosol optical properties were calculated using a look-up table as a function of wavelength and size parameter[45]. Here, we updated the refractive indices for mineral dust particles. The LW refractive indices were derived from averages of in situ measurements over 9 regions[12]. The dust radiative effect is estimated for each region and each size bin as the difference in the calculated radiative fluxes with all dust particles and with all dust particles except the size bin for the region being estimated in the calculation[46]. Thus, 5 simulations were conducted for each 9 regions with each refractive index. The results in Supplementary Figure 15 show the resulting radiative effects from the summation over each bin and region.

GISS Model E2.1. The distribution of dust aerosols and their radiative impact is calculated here using the One Moment Aerosol (OMA) version of the NASA Goddard Institute for Space Sciences Earth System ModelE2.1[47,48] that has horizontal resolution of 2° latitude by 2.5° longitude and 40 vertical layers that extend to 0.1 hPa, just above the stratopause. The dust

simulations described here are the same as analyzed by Kok et al.[3]. Further model description is given by Miller at al.[49] and Perlwitz et al.[50].

Dust sources are identified as arid lowlands[51], where dust emission increases with wind speed, while being inhibited by soil moisture[52]. Emission also increases with parameterized wind gustiness[53]. Transport occurs within five size classes (with diameters 0.1-2 μm, 2-4 μm, 4-8 μm, 8-16 μm and 16-32 μm, respectively). We did not use the largest bin (16-32 μm) because it exceeds the 20 μm maximum diameter used in the inverse model and instead generated a 16-20 μm bin based on the 8-16 μm bin and the GEOS/GOCART simulations, as described in Kok et al.[3]. The emitted ratio of clay (dust with $D < 2$ μm) and silt (dust with $D \geq 2$ μm) particles was prescribed to match retrievals of the aerosol size distribution at AERONET stations in dusty regions of high dust concentration, which resulted in a ratio consistent with measurements of the emitted size distribution compiled by Kok[32].

Model winds were nudged toward NCEP reanalysis values four times daily with a 1000-second relaxation time that was chosen to reproduce the magnitude of observed convergence. Dust is removed from the atmosphere by gravitational settling, turbulence within the surface layer and wet deposition. The latter includes below-cloud scavenging by precipitation with potential reevaporation, and in-cloud scavenging by nucleation, assuming that dust particles have a solubility of fifty percent, based upon explicit simulation of heterogeneous chemistry on dust particles[54].

The dust radiative effect is calculated from external mixtures of the size bins. The complex refractive index (CRI) for dust is prescribed at solar wavelengths assuming two equal external mixtures whose CRI is taken from retrievals by Sinyuk et al.[55] and Patterson et al.[56], respectively. At thermal wavelengths, the CRI is prescribed from measurements by Volz[14]. Longwave scattering is not explicitly calculated but its effect is approximated by increasing the total extinction by 30 percent[57], based upon calculations by Dufresne et al.[36]. Water coatings on dust particles by deliquescence and its radiative effect through particle radius is neglected.

The ModelE2.1 version of OMA represents only the first aerosol indirect effect[58], where aerosols influence cloud droplet number (CDN), which impacts cloud droplet size and optical thickness. The CDN at cloud base is specified from empirical relations based upon aerosol number and updraft speed[59]. The combined direct and indirect radiative effect of all aerosols in ModelE2.1 OMA is near -1 W/m2 in 2014[60], near the center of the range estimated by the Sixth Assessment Report of the Intergovernmental Panel on Climate Change[61].

Non-dust aerosols are prescribed in these simulations using monthly varying concentrations taken from separate OMA simulations of the CMIP6 historical period (1850-2014) with SST and sea ice prescribed from observations. This model version was subsequently found to have specified an erroneous particle radius for volcanic aerosols. While this distorted the stratospheric response following eruptions, the effect of this error on surface climate is small, as shown by comparisons with a corrected OMA version[60].

ModelE2.1 and other models in this study are used to calculate how clouds modify the clear-sky LW DRE, resulting in its all-sky counterpart. ModelE2.1 clouds are either convective or stratiform[62,63]. Convective clouds consist of two updrafts rising to their level of neutral buoyancy: one deep and undilute with the other diluted by entrainment of environmental air. Downdrafts created by detraining cloudy air and reevaporating moisture are also present[63]. The

optical depth of each cloud type depends upon condensed vapor, which is prognostic, along with precipitate[62,64]. While the areal fraction of each cloud type is calculated, radiative fluxes are computed assuming that the layer is either entirely cloudy or else clear. A random number from a uniform distribution between zero and one is generated at each time step for both convective and stratiform clouds and the cloud is assumed to impact radiation at all levels where its calculated fraction exceeds this number[65]. Thus, while radiative fluxes will differ at any single time step from those calculated assuming partial coverage, the climatological average will be the same because the partial coverage is emulated by the fractional occurrence of full coverage. This method of stochastic occurrence is computationally more efficient than assuming partial coverage because, in the former case, the radiative fluxes are calculated for only one grid box type: either clear or else cloudy.

GEOS/GOCART. Simulations performed with the Goddard Earth Observing System (GEOS) global Earth system model ran the Goddard Chemistry, Aerosol, Radiation, and Transport (GOCART) aerosol module [66,67]. GOCART simulates the dust particle size distribution in five non-interacting size bins (diameters: 0.2 – 2, 2 – 3.6, 3.6 – 6, 6 – 12, 12 – 20 μm). Dust emissions use an updated version of the scheme described in Ginoux et al.[51], where dust vertical flux is a function of the surface wind speed, soil moisture, and a topographically weighted source function. Emissions are distributed across our five size bins using the brittle fragmentation theory of Kok[68]. Dust optical properties are as described in Colarco et al.[69], assuming a spheroidal shape distribution and LW refractive indices compiled from various observational measurements as described in Koepke et al.[70] and synthesized in the OPAC database[16]. GEOS uses the Rapid Radiative Transfer Model for GCMs (RRTMG, Ref. [71]) for its LW internal radiative transfer, which uses 16 bands spanning 3.08 – 1000 μm in wavelength space. The GEOS implementation of RRTMG does not account for longwave scattering on aerosols, so that in addition to extinction due to gas absorption there is extinction also due to aerosol absorption. Prognostic water and ice clouds in the GEOS AGCM are from Bacmeister et al.[72] as modified with a sub-grid PDF distribution of humidity-related fields after Molod[73]. See Molod et al.[74] for additional details.

MONARCH. The Multiscale Online Non-hydrostatic AtmospheRe CHemistry (MONARCH) model, developed at the Barcelona Supercomputing Center[75-77], incorporates advanced chemistry and aerosol packages, including a comprehensive representation of the dust cycle. MONARCH is coupled online with the Non-hydrostatic Multiscale Model (NMMB)[78], providing a fully interactive framework for atmospheric composition and weather and climate simulations.

MONARCH employs the RRTMG (Rapid Radiative Transfer Model for GCMs) scheme[71] to compute shortwave and longwave radiative fluxes and associated heating rates using the correlated-k approach. The longwave component (RRTMG_LW) calculates fluxes across sixteen contiguous spectral bands spanning 3.08–1000 μm. Molecular absorbers considered in the model include water vapor, carbon dioxide, ozone, nitrous oxide, methane, oxygen, nitrogen, and several halocarbons (CFC-11, CFC-12, CFC-22, and CCl_4). Except for water vapor, which is computed online, all other gas concentrations are prescribed from climatological datasets in these simulations.

In RRTMG_LW, scattering is not explicitly modeled for either clouds or aerosols; instead, only extinction due to absorption is accounted for. The optical properties of clouds are parameterized per spectral band, following Hu and Stamnes[79] for water clouds and Fu et al.[80] for ice clouds.

Cloud fields are treated as grid-mean quantities without sub-grid variability, assuming a maximum-random cloud overlap configuration.

The dust module in MONARCH includes eight size transport bins, encompassing particles up to 20 µm in diameter, with the mass fraction of emitted dust in each bin parameterized following brittle fragmentation theory[32]. Dust particles are assumed to be externally mixed within each size bin, with no water uptake considered. While SW radiative interactions incorporate dust mineralogy-based refractive indices and non-spherical particle shapes, the LW component assumes spherical dust particles and utilizes refractive indices from the Optical Properties of Aerosols and Clouds (OPAC) dataset[16].

LMDZOR-INCA. Dust aerosol is represented by four modes in LMDZOR-INCA that cover aerosol diameters from 0.01 to 100 µm[81,82]. The prescribed size distribution of dust at emission is partitioned among the four modes (0.57%, 4.2%, 30.8%, 62.4%), which ensures consistency with Kok et al.[5] and measurements from the Fennec field campaign Experiment[83]. In the present study, the outputs from the dust simulations were reprojected on five bins up to a diameter of 20 µm as listed in Supplementary Table 4 and discussed in Kok et al.[3].

The radiative transfer code that describes the longwave portion of the spectrum consists of 16 bands with wavelengths that span from 3.33 to 1,000 µm. This radiative transfer code, the Rapid Radiative Transfer Model (RRTM), was developed at the European Centre for Medium-Range Weather Forecasts[84]. No corrections were applied to these results to account for scattering in the longwave, which is not accounted for in the radiative transfer code[85]. For each of these bands, optical parameters are read from lookup tables according to the particle diameter. Dust is considered externally mixed with regards to the aerosol components and has no affinity with water. The refractive index in the longwave spectrum was taken from the compilation of measurements from Di Biagio et al.[12]. Dust longwave radiative effects are computed for each band and each dust mode (or bin equivalent) of the size distribution through a double call to the radiation code, one in which dust is present and another one when dust concentrations are set to zero. Results are shown in Figure 2b for the summation of all five bins for the season and over the area for which measurements were reported.

Within LMDZ[86] cloud cover and cloud water content are computed using a statistical scheme using a lognormal function for deep convection[87] and a bigaussian function for shallow cumulus[88]. Cloud droplet and crystal number concentrations are diagnosed afterward for the radiation scheme only. In particular, the first indirect effect, due to soluble aerosols, is restricted to liquid clouds and to the liquid fraction of mixed clouds, whereas the effective sizes of ice crystals are those of the RRTM scheme as implemented in the ECMWF mode[86].

Correction of observed LW clear-sky DREE values to diurnally and seasonally averaged values.

Because the predictions of our data-driven analytical model are diurnally and seasonally averaged, we corrected measurements in our compilation of LW clear-sky DREE observations (Supplementary Table 5) as follows:

$$\widetilde{\Omega}_{\mathrm{obs},i} = \Omega_{\mathrm{obs},i} \frac{\widetilde{\Omega}_{\mathrm{mdl},i}}{\Omega_{\mathrm{mdl},i}}, \tag{10}$$

where $\Omega_{obs,i}$ denotes one of the published observationally based estimate (indexed by i) of the LW clear-sky DREE and $\widetilde{\Omega}_{obs,i}$ denotes its corresponding seasonally and diurnally averaged value (see Supplementary Table 5 and Fig. 1) using the correction factor $\widetilde{\Omega}_{mdl,i}/\Omega_{mdl,i}$. Here, $\widetilde{\Omega}_{mdl,i}$ is the diurnally averaged model result (plotted in Fig. 1) for the season (and location) that is the closest match to the time-of-year for which measurement i was made, whereas $\Omega_{mdl,i}$ is the model-calculated LW clear-sky DREE at the particular time-of-day and time-of-year for which the measurement was made. Its value was obtained by interpolating between the 6-hourly LW clear-sky DREE values calculated in our procedure using the 6-hourly reanalysis fields (see Methods). The correction factor $\widetilde{\Omega}_{mdl,i}/\Omega_{mdl,i}$ is substantially less than unity (of order ~0.80) for measurements made during daylight hours only[89-91]. This is because the LW DREE is largest in the middle of the day when both the surface temperature peaks and dust usually resides at higher altitude[90]. As such, correcting these observations to be representative of the diurnally averaged LW DREE is critical before comparison against (diurnally averaged) model results. Note that observationally based estimates of the LW clear-sky DREE are subject to numerous limitations[89,92], which we discuss below.

Limitations and caveats. We expect the calculation of the LW direct radiative effect and forcing with the data-driven analytical model to be more accurate than climate model results because of the explicit propagation of errors (see Supplementary Fig. 2), the inclusion of the spatiotemporally varying effects of LW scattering, the use of observationally constrained particle size distributions that include super coarse dust[3,22], and the integration of observational estimates of the LW DREE (Supplementary Fig. 2). Nonetheless, our methodology is subject to important limitations that could still cause possibly substantial biases. These limitations can roughly be divided into three groups: (1) uncertainties and limitations induced by simplifying assumptions that were made in order for the data-driven analytical model to remain analytically solvable, (2) errors in the data sets used in the data-driven analytical model, and (3) errors in the observational estimates of the LW clear-sky DREE.

The main errors and limitations of the analytical model are as follows. First, we assumed that $\tau_{LW} << 1$, allowing us to simplify the effects of dust on longwave radiation. However, when τ_{LW} becomes of order 1, multiple extinction effects start becoming important, which are not included, possibly causing a slight underestimate of the LW radiative effects. This could affect the calculated LW DRE at the dustiest locations, as the maximum seasonally averaged LW DAOD reaches ~0.5 in spring and summer (and ~0.3 in fall and winter) (Supplementary Fig. 11). Moreover, the LW DAOD on an event basis could reach substantially above the seasonal average, contributing to a further underestimation of the LW DRE. Second, we similarly assumed that atmospheric absorption is small (i.e., $\bar{\epsilon}_{bel}$, $\bar{\epsilon}_{abv} << 1$). Here also, the maximum seasonally averaged $\bar{\epsilon}_{bel}$ and $\bar{\epsilon}_{abv}$ reach ~0.5 in summer, such that our assumption that $\bar{\epsilon}_{bel}$ and $\bar{\epsilon}_{abv} << 1$ would cause a slight underestimation of the LW DRE. Third, we simplified the LW radiative effects by using optical and radiative properties (e.g., $\bar{\epsilon}_{abv}$, $\bar{\omega}_b$, $\bar{\beta}_{\downarrow,b}$, $\bar{k}_{ext,b}$, $\bar{\tau}_{LW}$) averaged over the entire atmospheric window (8 – 14 μm). However, optical properties of both dust and atmospheric absorption can vary substantially in this spectral range[12,93]. As such, this simplified treatment of radiative effects will cause errors for effects that are non-linear in the spectrally averaged optical and radiative properties. Examples of these include optical properties (Supplementary Table 4), which can have a non-linear dependence on wavelength[93], the occurrence of dust optical depth or atmospheric absorptivity of order 1 for parts of the spectrum,

for which the radiative effects become sub-linear in the optical depth or absorptivity (see previous two limitations). Moreover, any effects that are non-linear in the spectrally averaged optical and radiative properties, such as the dependence of mass absorption efficiency with complex refractive index for large values of the imaginary refractive index, will cause errors. Fourth, because our analysis is done with seasonally averaged variables, we neglect any sub-seasonal co-variability of LW DAOD with temperature and humidity profiles and ozone concentrations. Fifth, although we use observational estimates of the LW DREE to constrain the results of our data-driven analytical model, the agreement of our results with observations does not necessarily imply that all of the relevant processes have been accurately captured or that each parameter value used is realistic. Indeed, given the remaining uncertainties in dust LW optical properties, dust altitude, and dust size distribution, it is likely that some of the bootstrap iterations achieve RMSE < $RMSE_{max}$ due to compensating errors among parameters. This phenomenon, known as "equifinality", highlights that different parameter combinations can produce similar model outputs, a well know result in perturbed parameter ensemble model study approaches[94]. Sixth, our analysis assumes that dust produces zero top-of-atmosphere direct radiative effect outside of the atmospheric window because of high absorptivity outside of that spectral region, primarily due to absorption by water vapor. However, for high and cold dust layers, the overhead absorptivity might be small enough that dust still exerts some direct radiative effect outside of the atmospheric window, causing some overestimation of the LW DRE. Seventh, our analysis does not account for the possible enhancement of LW extinction created by coatings on supermicron dust particles[95,96]. And finally, our calculation of the LW DRF assumed that the LW DREE has stayed constant over time since the pre-industrial period. However, surface and atmospheric temperatures, and concentrations of water vapor, CO2, and other greenhouse gases have changed from the pre-industrial to the historical period. Additionally, the vertical profile and horizontal distribution of dust has likely changed[97]. All these changes modify the LW DREE, which we do not account for.

In addition to these limitations inherent in the analytical model, there are also several important limitations on the data used to drive the analytical model. The DustCOMM dust climatology that is an important input to the analytical model has many experimental, observational, and modeling uncertainties propagated, but might still be subject to important biases, as discussed in Kok et al.[3,4]. One especially important such bias for the LW DRE is that the vertical profile of dust in DustCOMM is based on an ensemble of model simulations, but these are known to struggle to reproduce the vertical profile of dust[8,98]. A second important limitation on the data driving the analytical model is the large uncertainty in the size-dependent LW optical properties of dust. Although we used a series of LW refractive index data sets[12,13,15,16], and thus propagated the uncertainty quantified by the spread in these data sets, this ensemble of refractive indices might still be biased because of various factors, including the limited number of dust samples analyzed, the inherent difficulties of reproducing natural dust emission in a laboratory[99], experimental limitations and biases in measuring LW optical properties[12], and the need to represent all dust with a single set of LW optical properties, whereas in reality there exists (poorly quantified) regional variability due to changes in mineralogy[12,46]. A third important limitation on the data driving the analytical model is that the ratio of the all-sky to clear-sky LW DRE that we used from an ensemble of model simulations (Supplementary Fig. 6) might be biased because these simulations do not account for LW scattering, which responds differently to clouds below the dust layer than does LW absorption. A fourth limitation is that the analytical

model uses only a single reanalysis data set (MERRA-2) to provide the atmospheric properties and surface temperature and thus does not account for uncertainty in these variables.

Note that the effects of all the above limitations on the calculated LW direct radiative effect and forcing are mitigated by our procedure of retaining only the subset of bootstrap iterations that are consistent with observational LW DREE estimates (explained above and shown in Supplementary Figs. 2 and 3). However, those observational estimates have important limitations themselves. First, studies that used ground-based or in situ measurements to estimate the LW DREE necessarily mostly focused on (intense) dust events, which might have different properties (e.g., dust vertical profile and size distribution) and atmospheric conditions (e.g., drier) than the seasonally averaged properties used to drive the analytical model. Second, estimates of the LW DREE over land that used observed temperatures[91,100] inherently include the fast response of the surface temperature to the dust loading. Since dust on balance decreases surface temperatures by decreasing incident solar radiation, this effect reduces the upwelling LW flux, which is then erroneously interpreted as an instantaneous LW dust radiative effect. This effect can cause an overestimate of the instantaneous LW DREE that is at most 25-35%[92]. Third, satellite-based studies usually assume that the LW DRE is zero when clouds are present in the atmospheric column[89]. This is reasonable when dust is located below clouds, but dust still exerts a LW cooling effect when located above clouds, which is neglected. Fourth, errors in cloud screening can cause LW effects of clouds to be attributed to dust or vice versa[89]. Similarly, errors in aerosol typing can affect the calculation of the dust aerosol optical depth. And fifth, most of the LW DREE observations were obtained for North African dust (Fig. 1) in Spring and Summer, such that systematic differences in dust LW optical properties with season and location that are not captured by the analytical model could induce a bias.

Supplementary Tables

Supplementary Table 1. Compilation of global model simulations of the global annual mean LW DRE at TOA.

Study	Model	Annual global LW clear-sky DRE (Wm^{-2})	Annual global LW all-sky DRE (Wm^{-2})	All-sky LW DRE due to scattering (Wm^{-2})
This study	CESM/CAM4	0.26[a]	0.20[a]	0.07
This study	IMPACT	0.19	0.13	0
This study	GISS ModelE2.1	0.13[b]	0.10[b]	0.02
This study	GEOS/GOCART	0.12	0.10	0
This study	MONARCH	0.17	0.13	0
This study	INCA	0.15	0.12	0
Heald et al. (2014)[101]	GEOS-Chem–RRTMG	0.16	0.14	0
Albani et al. (2014)[44]	CESM/CAM4	--	0.125[c]	0
Albani et al. (2014)[44]	CESM/CAM5	--	0.14	0
Woodage & Woodward (2014)[102]	UK HiGEM	--	0.32	0.16[d]
Scanza et al. (2015)[103]	CESM/CAM4	--	0.09	0
Scanza et al. (2015)[103]	CESM/CAM5	--	0.13	0
Klingmuller et al. (2019)[104]	ECHAM/MESSy	--	0.09	0
Tucella et al. (2020)[105]	GEOS-Chem	--	0.09	0
Di Biagio et al. (2020)[81]	LMDZOR-INCA	--	0.22[e]	0.11
Checa-Garcia et al. (2021)[82]	LMDZOR-INCA	0.14	--	--
Ito et al. (2021)[46]	IMPACT	--	0.23[e]	0.12
Li et al. (2021)[106]	CESM/CAM5	--	0.11[a]	0.04
Li et al. (2021)[106]	CESM/CAM6	--	0.14[a]	0.05
Li et al. (2021)[106]	MONARCH	--	0.17	0
Woodward et al. (2022)[107]	HadGEM3-GC3.1	--	0.164[d]	0.082[d]
Woodward et al. (2022)[107]	UKESM1		0.194[d]	0.097[d]
Feng et al. (2022)[108]	E3SMv1	--	0.12f	0
Ke et al. (2022)[109]	CESM/CAM5/MAM9	--	0.13	0
Wang et al. (2024)[110]	SPRINTARS	--	0.16[g]	0
--	--	0.16	0.13 (0.09 – 0.23)[h]	0.03

[a]The effect of longwave scattering was approximated by increasing the LW DRE at the top-of-atmosphere by 50%.
[b]The effect of longwave scattering was approximated by increasing the LW extinction due to dust by 30%.
[c]Mean of the C4wn and C4fn model simulations.
[d]Study included the effect of longwave scattering but did not report the fraction of the top-of-atmosphere LW DRE that was contributed by scattering, which we therefore assumed here to be 50%.
[e]The effect of longwave scattering was approximated by scaling the LW DRE at the top-of-atmosphere by a factor of 2.04.
[f]Mean of five model simulations with different resolution, dust size distribution, and model parameter settings.
[g]Mean of three model simulations with different optical properties and dust size distribution.
[h]Reported here is the median and the 90% confidence interval (CI) of the 24 studies. The CI was obtained by eliminating the lowest and highest values, leaving the 22 central values of the 24 model results, which corresponds approximately to the central 90% of model results.

Supplementary Table 2. Size-resolved dust radiative effects. Listed are the aerosol optical depth in both the LW (averaged over the 8-14 μm spectral range) and the SW (550 nm) spectra, the TOA DRE for both all-sky and clear sky conditions, and the LW direct radiative effect efficiency (DREE). All values represent global annual means and were obtained from the analytical model.

Diameter range	LW DAOD ($\times 10^{-3}$)	SW DAOD	LW clear-sky DRE (Wm^{-2})	LW all-sky DRE (Wm^{-2})	LW DREE ($Wm^{-2}\,\tau_{SW}^{-1}$)

$D \leq 2.5$ μm	1.1	0.013	0.04	0.03	1.4
$2.5 < D \leq 5$ μm	2.7	0.007	0.10	0.07	14
$5 < D \leq 10$ μm	4.3	0.004	0.12	0.10	30
$10 < D \leq 20$ μm	2.5	0.002	0.05	0.04	20
$D > 20$ μm	0.4	0.0004	0.01	0.01	10
All dust	11.0 ± 3.1	0.027 ± 0.005	0.32 ± 0.08	0.25 ± 0.06	11 ± 5

Supplementary Table 3. Statistical parameters quantifying agreement of six global models with LW DREE observations.

Global model	Annual global LW all-sky DRE (Wm^{-2})	R^2	RMSE ($Wm^{-2}\ \tau_{SW}^{-1}$)	Bias ($Wm^{-2}\tau_{SW}^{-1}$)
CESM/CAM4	0.20	0.21	5.2	-4.4
IMPACT	0.13	0.35	9.0	-8.7
GISS ModelE2.1	0.10	0.48	6.5	-6.1
GEOS/GOCART	0.10	0.00	8.4	-7.7
MONARCH	0.13	0.25	7.2	-6.8
LMDZOR-INCA	0.12	0.07	9.7	-9.3

Supplementary Table 4. Optical properties in the atmospheric window (i.e., averaged over the 8-14 μm spectral range) for each dust particle bin of each of the six different model simulations used in the DustCOMM dust climatology (see Ref. [3]). Reported values represent the average and standard deviation. The variability in the optical properties for a given particle bin is due to variability in the sub-bin dust size distribution, which is based on Adebiyi and Kok[1] [see Supplementary Equations. (7)-(9)], and in the dust complex refractive index in the atmospheric window, which is based on a random drawing of one of six data sets (see Supplementary Table 6).

Model	Bin number	Diameter range (μm)	SSA ($\bar{\omega}_b$)	Downscatter fraction ($\bar{\beta}_{\downarrow,b}$)	Mass ext. efficiency ($\bar{k}_{\mathrm{ext},b}$ in m^2g^{-1})
CESM/CAM4	1	0.1-1	0.014 ± 0.006	0.495 ± 0.001	0.084 ± 0.036
	2	1-2.5	0.139 ± 0.055	0.472 ± 0.004	0.106 ± 0.040
	3	2.5-5	0.39 ± 0.13	0.374 ± 0.016	0.181 ± 0.078
	4	5-10	0.52 ± 0.15	0.231 ± 0.005	0.203 ± 0.057
	5	10-20[a]	0.52 ± 0.10	0.150 ± 0.009	0.118 ± 0.022
IMPACT	1	0.1-1.26	0.026 ± 0.011	0.493 ± 0.001	0.086 ± 0.036
	2	1.26-2.5	0.150 ± 0.059	0.470 ± 0.004	0.109 ± 0.041
	3	2.5-5	0.39 ± 0.13	0.374 ± 0.016	0.181 ± 0.078
	4	5-20	0.52 ± 0.12	0.200 ± 0.009	0.159 ± 0.036
GISS ModelE2.1	1	0.2–0.36	$(0.9 \pm 0.4)\times10^{-3}$	0.499 ± 0.001	0.081 ± 0.036
	2	0.36-0.6	$(3.9 \pm 1.6)\times10^{-3}$	0.498 ± 0.002	0.082 ± 0.036
	3	0.6-1.2	0.025 ± 0.010	0.493 ± 0.008	0.086 ± 0.036
	4	1.2-2	0.103 ± 0.041	0.479 ± 0.003	0.100 ± 0.039
	5	2-4	0.32 ± 0.12	0.417 ± 0.013	0.156 ± 0.065
	6	4-8	0.50 ± 0.14	0.267 ± 0.009	0.212 ± 0.072
	7	8-16	0.53 ± 0.12	0.169 ± 0.007	0.146 ± 0.030
	8	16-20	0.49 ± 0.05	0.128 ± 0.012	0.084 ± 0.011
GEOS/GOCART & LMDZOR-INCA[b]	1	0.2–2	0.08 ± 0.03	0.483 ± 0.002	0.095 ± 0.038
	2	2-3.6	0.230 ± 0.11	0.430 ± 0.010	0.148 ± 0.061

	3	3.6-6	0.46 ± 0.15	0.310 ± 0.014	0.210 ± 0.085
	4	6-12	0.54 ± 0.14	0.204 ± 0.004	0.185 ± 0.044
	5	12-20	0.51 ± 0.08	0.140 ± 0.009	0.103 ± 0.017
MONARCH	1	0.2–0.36	$(0.9 \pm 0.4)\times10^{-3}$	0.499 ± 0.001	0.081 ± 0.036
	2	0.36-0.6	$(3.9 \pm 1.6)\times10^{-3}$	0.498 ± 0.002	0.082 ± 0.036
	3	0.6-1.2	0.025 ± 0.010	0.493 ± 0.008	0.086 ± 0.036
	4	1.2-2	0.103 ± 0.041	0.479 ± 0.003	0.100 ± 0.039
	5	2-3.6	0.30 ± 0.11	0.430 ± 0.010	0.148 ± 0.061
	6	3.6-6	0.46 ± 0.15	0.310 ± 0.014	0.210 ± 0.085
	7	6-12	0.54 ± 0.14	0.204 ± 0.004	0.185 ± 0.044
	8	12-20	0.51 ± 0.08	0.140 ± 0.009	0.103 ± 0.017
All models	SC1[c]	20-35	0.50	0.075	0.084
	SC2[c]	35-62.5	0.50	0.075	0.044
	SC3[c]	62.5-100	0.50	0.075	0.024

[a]Denotes an additional bin added to the original model output in order to extend the particle diameter range to 20 µm. See Kok et al. [3] for details.

[b]Both models use the same particle bins. See Kok et al. [3] for details.

[c]Results for each model were extended with three bins to include dust with diameters between 20 to 100 µm, using simulations with the Community Earth System Model (CESM) from Meng et al. [10], as described in Methods.

Supplementary Table 5. Compilation of observational estimates of the LW clear-sky direct radiative effect efficiency (DREE) at the top-of-atmosphere. Listed are the originally reported ($\Omega_{\mathrm{obs},i}$), the diurnally corrected, and the diurnally and seasonally corrected ($\tilde{\Omega}_{\mathrm{obs},i}$) values of the LW DREE (see Supplementary Equation 10), in units of Wm^{-2} per unit of SW (550 nm) optical depth. Also listed is whether the study was primarily based on in situ data or on satellite data and whether the observational estimate was representative of the LW DREE over land or over ocean.

**Reference**	**Study area & coordinates**	**Season or months**	**Study type**	**Time (UTC)**	**Reported LW DREE**	**Diurnal LW DREE**	**Diurnal & seasonal LW DREE**
Highwood et al. (2003)[111]	Between Sal Island and Daqar (16 °N, ~20 °W)	September	In situ; over ocean	Diurnal	9.7	9.7	9.6
Brindley and Russell (2009)[89]	West Africa (16 - 28 °N, 16 - 4 °W)	MAM	Satellite; over land	0800-1600	18	14.2	14.2
Brindley and Russell (2009)[89]	West Africa (16 - 28 °N, 16 - 4 °W)	JJA	Satellite; over land	0800-1600	17	14.2	14.2
Brindley and Russell (2009)[89]	Niger/Chad (15 - 20 °N, 5 - 20 °E)	MAM	Satellite; over land	0800-1600	15	11.6	11.6
Brindley and Russell (2009)[89]	Niger/Chad (15 - 20 °N, 5 - 20 °E)	JJA	Satellite; over land	0800-1600	16	13.1	13.1
Brindley and Russell (2009)[89]	Sudan (15 - 22 °N, 22 - 36 °E)	MAM	Satellite; over land	0800-1600	19	14.9	14.9
Brindley and Russell (2009)[89]	Sudan (15 - 22 °N, 22 - 36 °E)	JJA	Satellite; over land	0800-1600	21	17.4	17.4
Brindley and Russell (2009)[89]	Egypt/Israel (23 - 32 °N, 25 - 35 °E)	MAM	Satellite; over land	0800-1600	19	16.0	16.0
Brindley and Russell (2009)[89]	Egypt/Israel (23 - 32 °N, 25 - 35 °E)	JJA	Satellite; over land	0800-1600	25	21.1	21.1
Brindley and Russell (2009)[89]	North Libya (27 - 33 °N, 15 - 25 °E)	MAM	Satellite; over land	0800-1600	18	14.8	14.8
Brindley and Russell (2009)[89]	North Libya (27 - 33 °N, 15 - 25 °E)	JJA	Satellite; over land	0800-1600	20	16.6	16.6
Brindley and Russell (2009)[89]	South Libya (23 - 27 °N, 15 - 25 °E)	MAM	Satellite; over land	0800-1600	16	12.5	12.5
Brindley and Russell (2009)[89]	South Libya (23 - 27 °N, 15 - 25 °E)	JJA	Satellite; over land	0800-1600	18	14.5	14.5

Yang et al. (2009)[90]	Sahara (15 - 30 °N, 10 °W - 30 °E)	JJAS	Satellite; over land	1030 and 1330	18.5	14.4	15.0
Xia and Zong (2009)[91]	Taklimakan desert (36 - 42 °N, 75 - 95 °E)	May	Satellite; over land	0500	28.4	21.0	19.1
Hansell et al. (2010)[112]	Cape Verde (16.73 °N, 22.93 °W)	September	In situ; over land	Diurnal (ocean)	13	13	12.8
Osborne et al. (2011)[100]	Mauritania and Niger (18 °N, 6.45 °W)	June	In situ; over land	Diurnal	17.2	17.2	18.5
Hansell et al. (2012)[113]	East of Taklimakan desert (39 °N, 101 °E)	April and May	In situ; over land	Diurnal	19	19	18.4
Di Sarra et al. (2011)[114] & Meloni et al. (2015)[115]	Lampedusa (35.5 °N, 12.6 °W)	March and May	In situ; over ocean	Diurnal	10.3	10.3	10.3
Meloni et al. (2018)[116]	Lampedusa (35.5 °N, 12.6 °W)	June	In situ; over ocean	Diurnal	15.8	15.8	16.3
Song et al. (2018)[117]	Tropical North Atlantic (10 - 30 °N, 45 - 20 °W)	JJA	Satellite; over ocean	Diurnal	10.5	10.5	10.5

Supplementary Table 6. Values of the six atmospheric window-averaged (8-14 μm) complex refractive indices used in this study.

Reference	Complex refractive index
Volz (1972)[13]	1.54 + 0.11*i*
Volz (1973)[14]	1.83 + 0.33*i*
Fouquart et al. (1987)[15]	1.00 + 0.28*i*
Hess et al. (1998)[16]	1.85 + 0.35*i*
Di Biagio et al. (2014)[17]	1.59 + 0.17*i*
Di Biagio et al. (2017)[12]	1.45 + 0.12*i*

Supplementary Figures

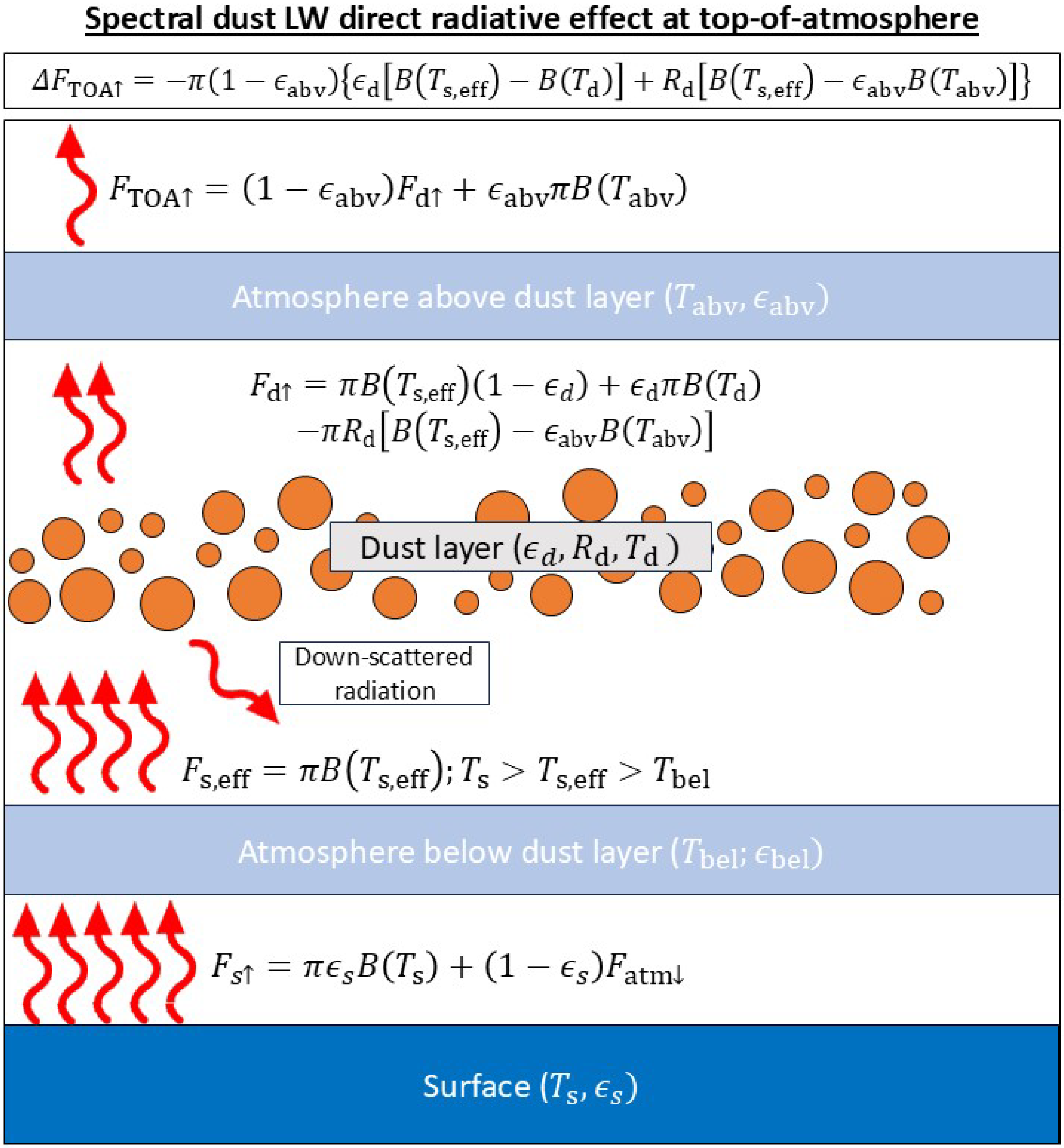

Supplementary Figure 1. Conceptual model of the longwave (LW) direct radiative effect (DRE) at the top-of-atmosphere (TOA) created by a dust layer. The dependence of most variables on the wavelength λ is not explicitly denoted for simplicity but is defined in the text.

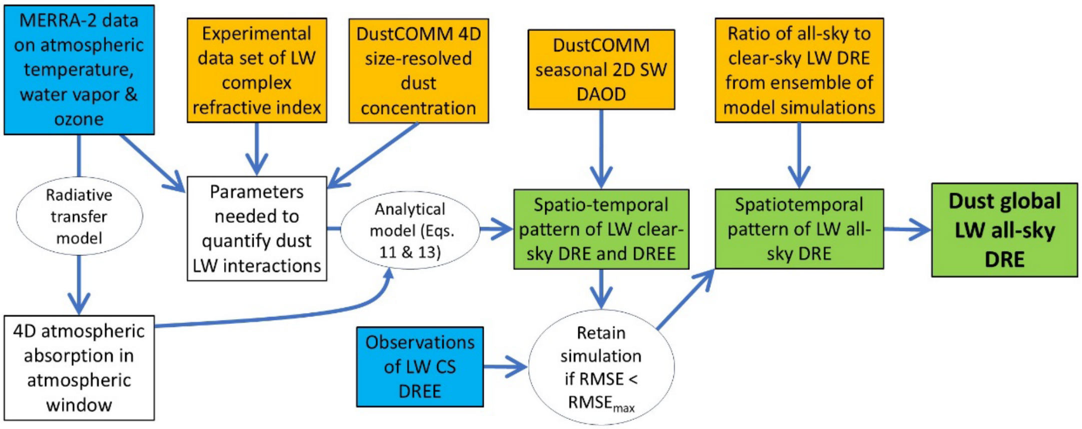

Supplementary Figure 2. Schematic overview of the methodology used to constrain the longwave (LW) all-sky direct radiative effect (DRE) and direct radiative forcing (DRF) at top-of-atmosphere. Orange boxes denote data from which different random realizations are drawn for each bootstrap iteration, blue boxes denote inputs of observationally informed data that are the same for each bootstrap iteration, and green boxes denote results that are reported in figures in the main text.

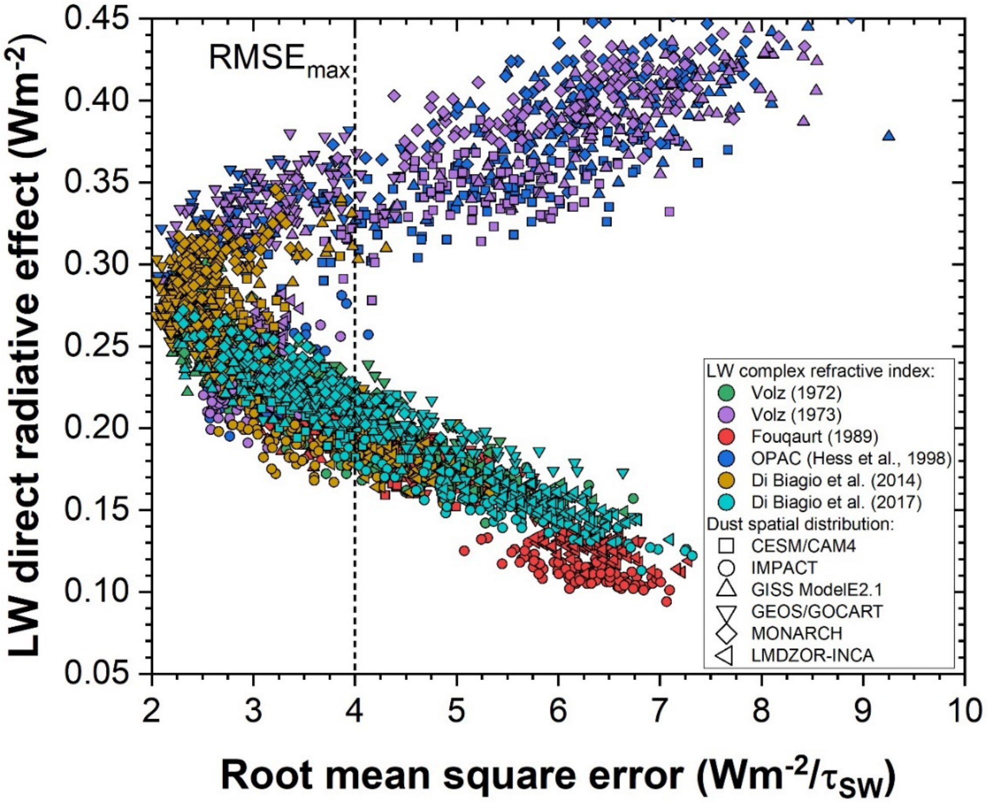

Supplementary Figure 3. Root mean square error of individual bootstrap iterations of the data-driven analytical model with respect to a compilation of observational estimates of the dust longwave (LW) direct radiative effect efficiency. Shown are 100 individual bootstrap iterations for each combination of the six LW complex refractive indices (Supplementary Table 5; distinguished by symbol color) and the six global model simulations that co-determine the dust spatial distribution (see Kok et al.[1]; distinguished by symbol type).

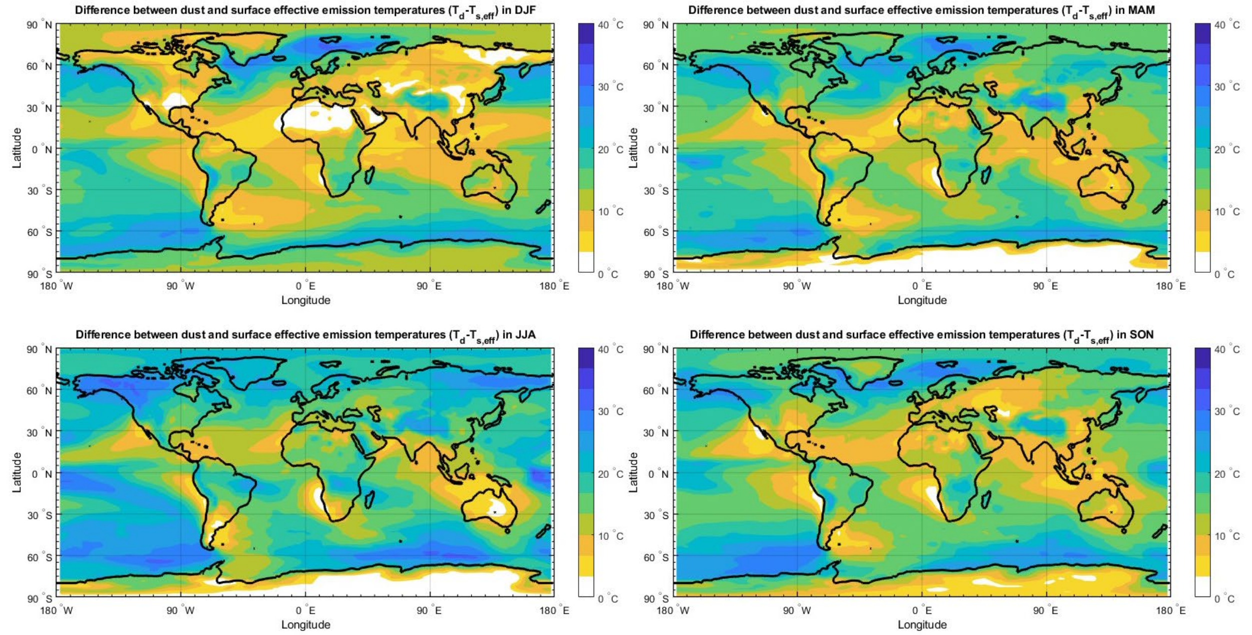

Supplementary Figure 4. Difference between the dust layer temperature (T_{d}) and the effective surface emission temperature (T_{bel}) as a function of season.

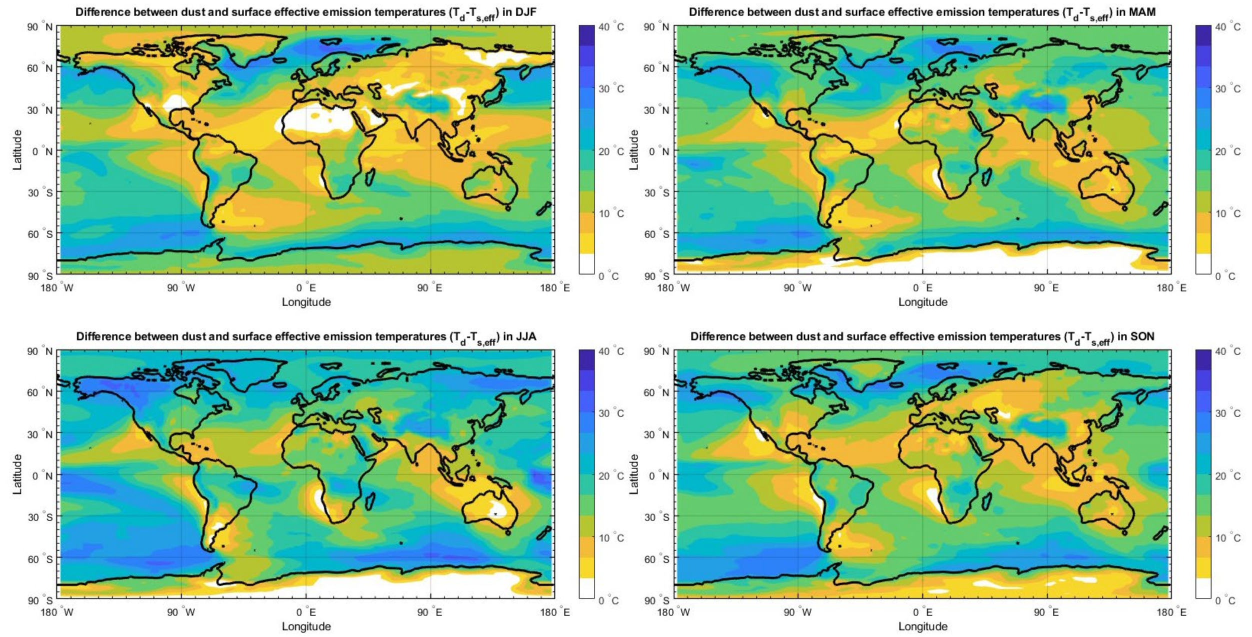

Supplementary Figure 5. Elevation of dust layer centroid above the local surface. The dust layer centroid is calculated as $z_{\mathrm{d}} = \int_0^\infty \bar{\beta}_{\mathrm{LW}}(z) z dz \,/\, \int_0^\infty \bar{\beta}_{\mathrm{LW}}(z) dz$, where the volume extinction coefficient in the LW spectrum is calculated as $\bar{\beta}_{\mathrm{LW}}(z) = \sum_b^{n_b} \rho_b(z) \bar{k}_{\mathrm{ext},b}$, where the index b sums over the n_{b} particle size bins, ρ_b is the density (kgm^{-3}) of dust in bin b in the layer, and $\bar{k}_{\mathrm{ext},b}$ is the mass extinction efficiency of bin b (Supplementary Table 4).

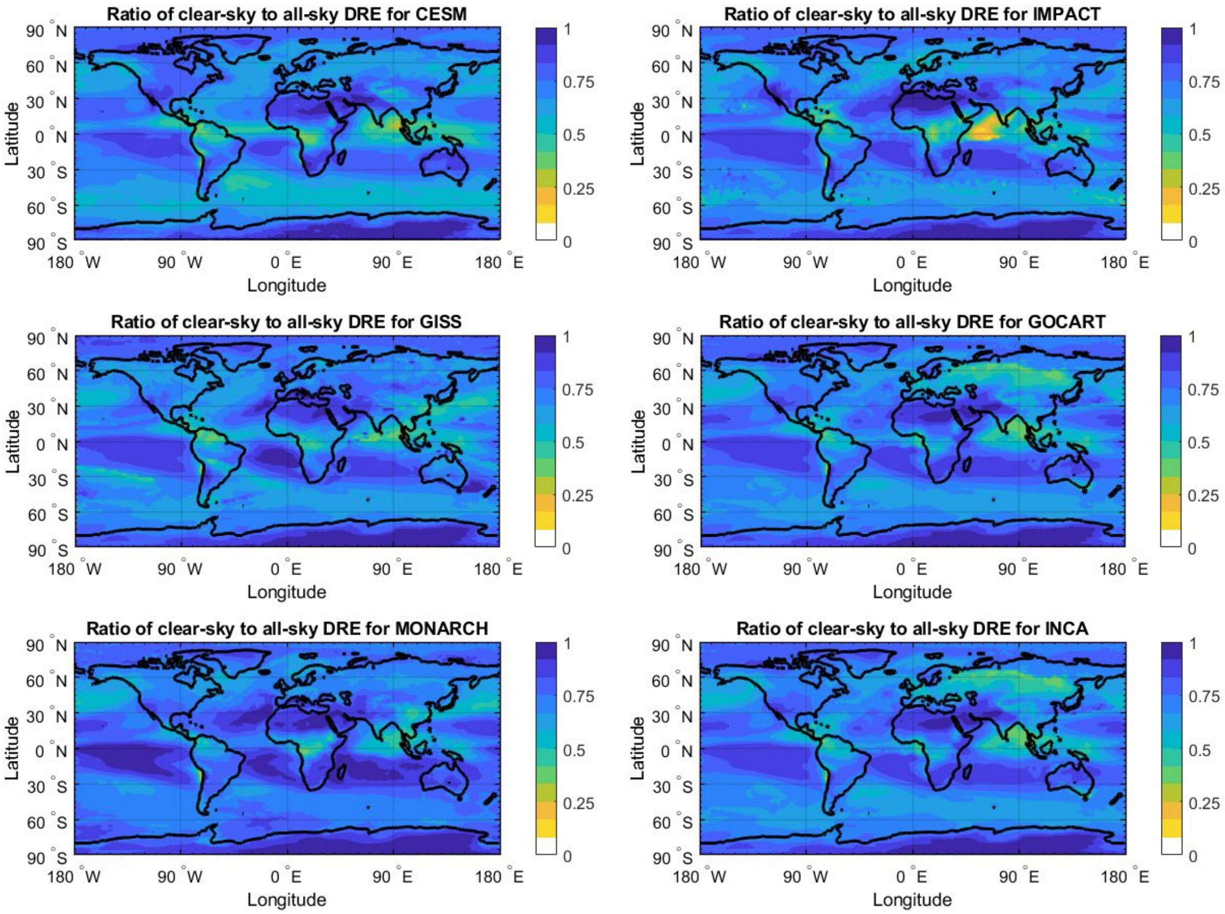

Supplementary Figure 6. Maps of the ratio of the clear-sky to the all-sky longwave (LW) direct radiative effect (DRE) at the top-of-atmosphere for the six model simulations in our ensemble.

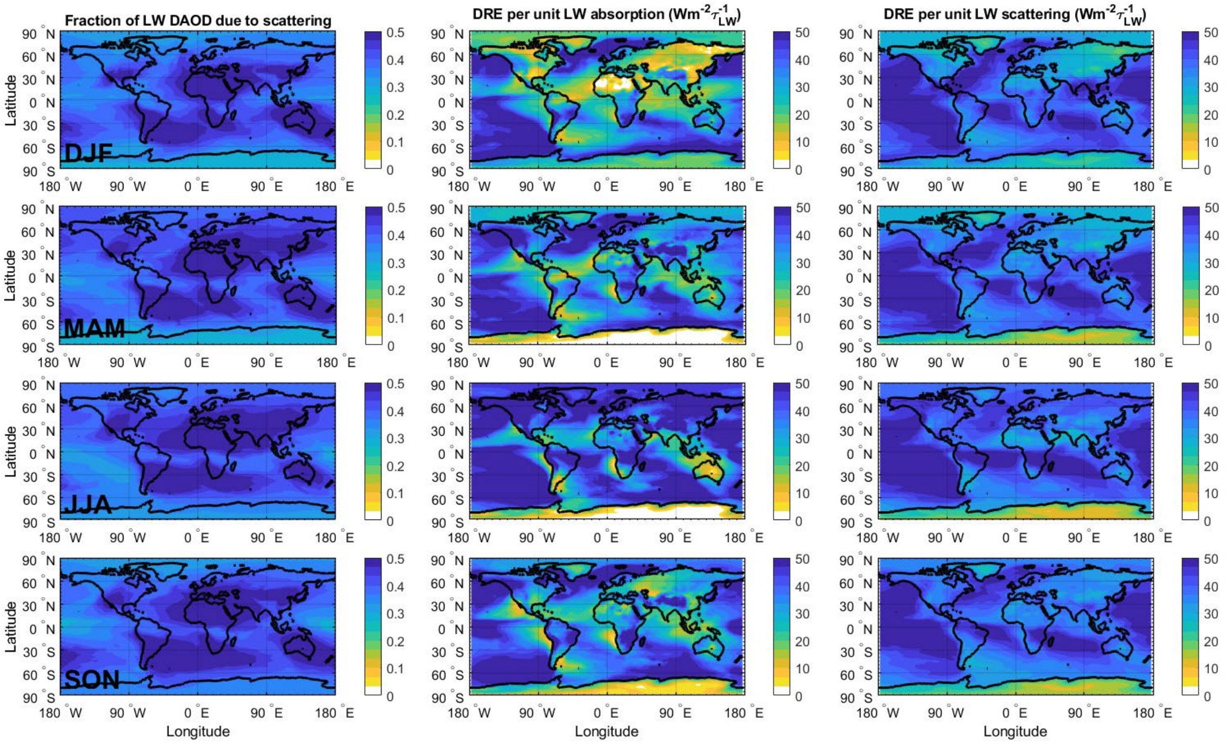

Supplementary Figure 7. Separation of seasonal top-of-atmosphere (TOA) longwave (LW) radiative effects into contributions from absorption and scattering. The fractional contribution of scattering to the dust aerosol optical depth (τ_{LW}) in the atmospheric window (averaged across 8-14 μm) is somewhat below 0.5 in dust source regions, decreasing to approximately 0.4 in remote regions (left columns). However, because 28 ± 3 % of scattering interactions result in downscattering (Supplementary Fig. 8), scattering is relatively effective in perturbing Earth's radiative energy budget, with a unit of longwave (LW) dust aerosol optical depth (DAOD) from scattering (right column) generating a greater TOA radiative effect than a unit of LW DAOD from absorption (middle column). In the global annual mean, a unit of LW DAOD due to scattering generates 33 ± 7 Wm^{-2} of TOA radiative effect, whereas a unit of LW DAOD from absorption generates 26 ± 7 Wm^{-2}, which is less than 80% of the radiative effect produced by scattering.

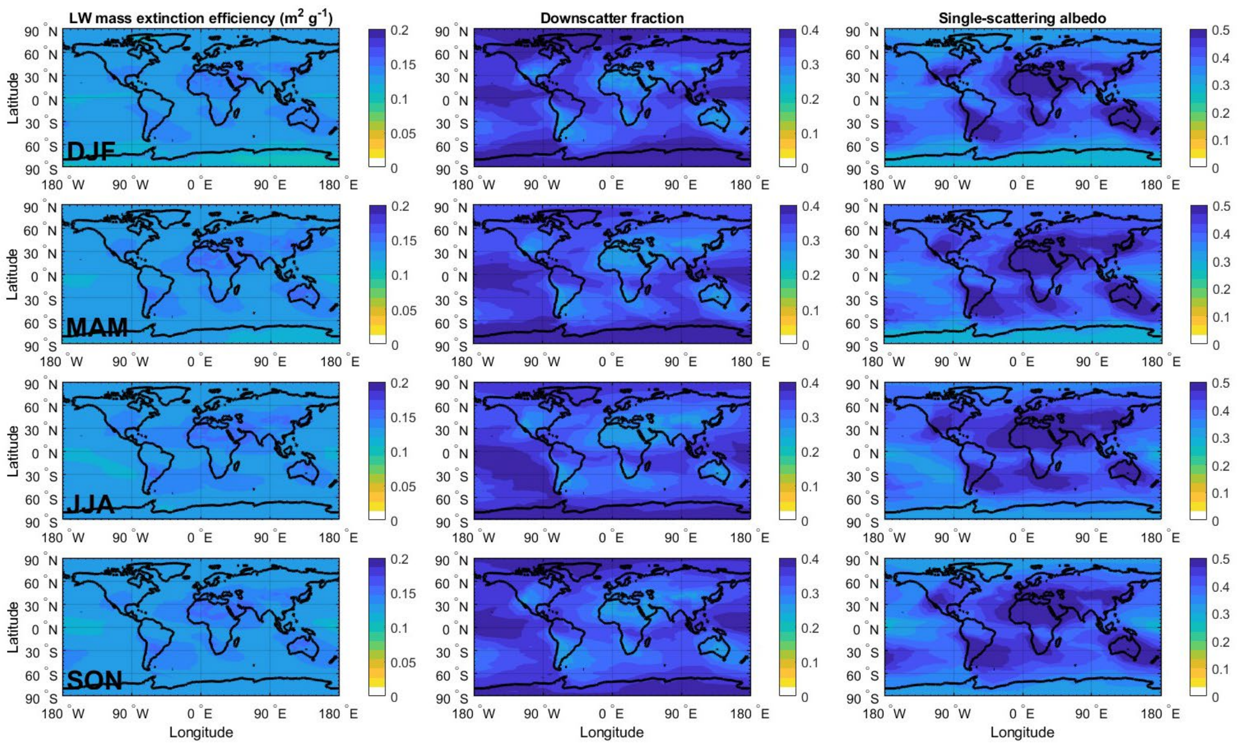

Supplementary Figure 8. Spatial variability of dust optical properties in the longwave (LW) spectrum. Shown are the seasonally averaged mass extinction efficiency $\bar{k}_{\mathrm{ext}}$ (left column), downscatter fraction ($\bar{\beta}_{\downarrow}$; middle column), and single-scattering albedo ($\bar{\omega}$; right column), averaged over the atmospheric window (8-14 μm).

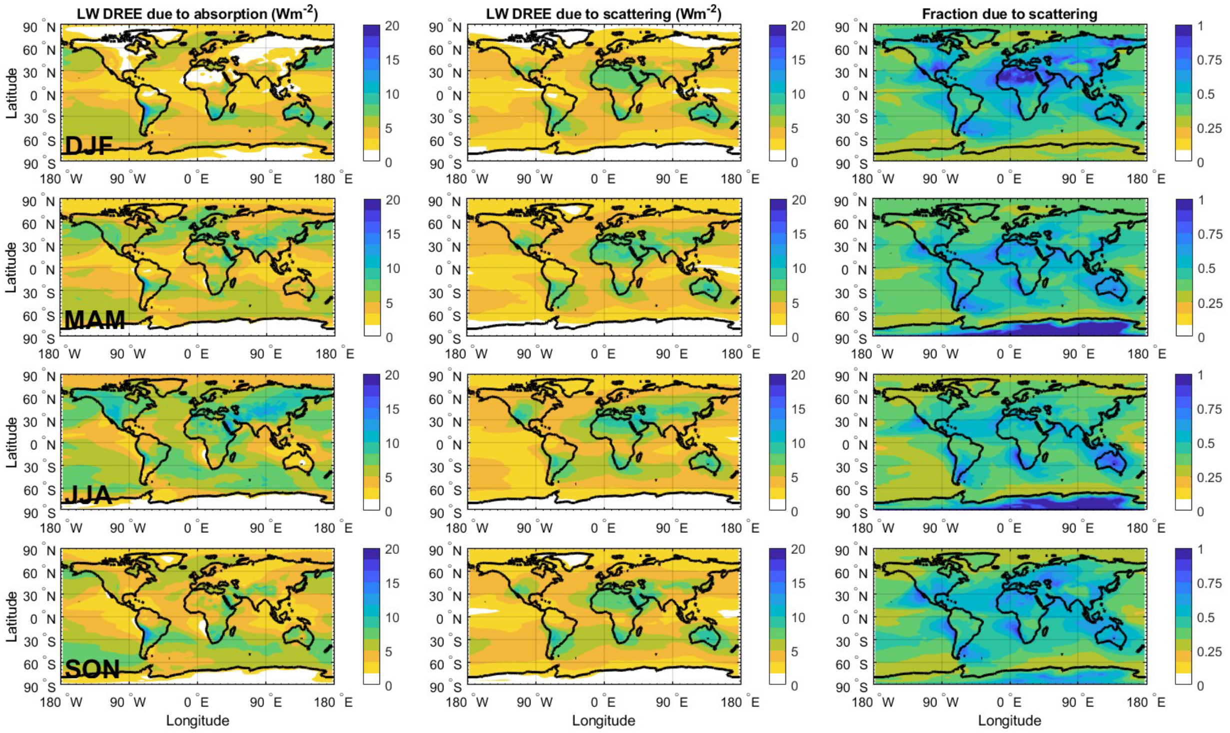

Supplementary Figure 9. The longwave (LW) direct radiative effect efficiency (DREE) at the top-of-atmosphere that is due to absorption (left column), due to scattering (middle column), and the fraction of the LW DREE warming that is due to scattering (right column), all as a function of season. The spatial pattern in the LW absorption DREE is primarily due to the spatiotemporal pattern of the difference in the emission temperatures of dust and the surface (Supplementary Fig. 4), which in turn depends largely on the dust altitude (Supplementary Fig. 5). In contrast, the spatial pattern in the LW scattering DREE does not depend on the temperature of the dust layer and instead is primarily determined by the spatial patterns of the single-scattering albedo, the downscatter fraction (Supplementary Fig. 8), and overhead atmospheric absorption (Supplementary Fig. 12g). The correlation between the spatially resolved LW DREE due to absorption and scattering is modest, with Pearson correlation coefficients of 0.61, 0.70, 0.78, and 0.64 for DJF, MAM, JJA, and SON, respectively (0.69 for the annual mean results). Note that the fractional contribution due to scattering is very large in polar winter because the presence of persistent temperature inversions reduces the warming effect of dust absorption of LW radiation, even to the point that it produces net cooling. All results are for clear-sky conditions.

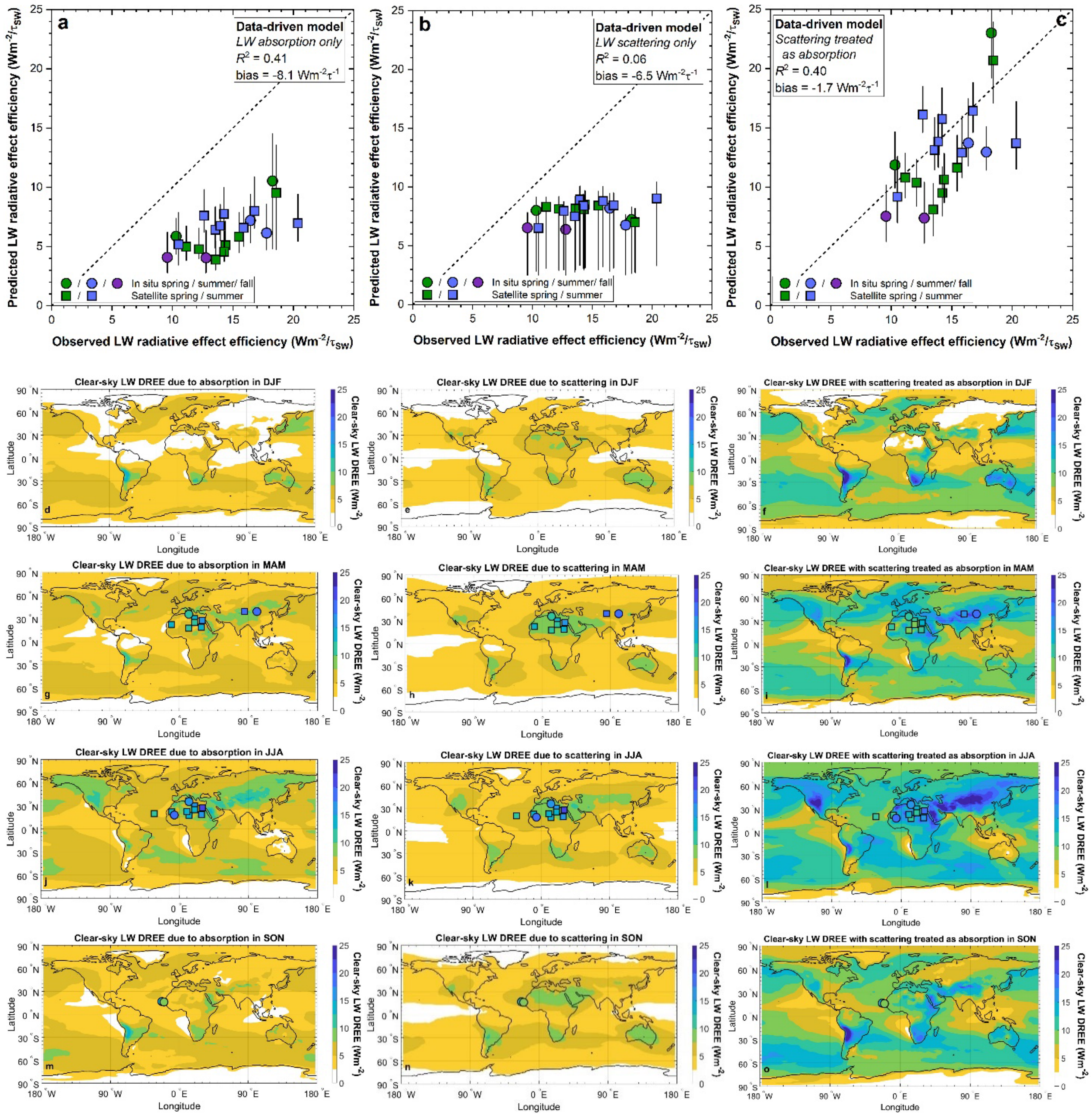

Supplementary Figure 10. Analytical model predictions of clear-sky longwave (LW) direct radiative effect efficiency (DREE) due only to absorption interactions (left column), due only to scattering interactions (middle column), and with scattering treated as absorption by setting the single-scattering albedo equal to zero (right column). Shown for each of these three cases are the comparison against observational estimates of LW clear-sky DREE derived mainly from in situ (colored circles) and satellite (colored squares) data (panels **a-c**). Also shown are the spatial patterns of the LW clear-sky DREE for each of the three cases, along with the observational constraints, for boreal winter (DJF; panels **d-f**), boreal spring (MAM; panels **g-i**), boreal summer (JJA; panels **j-l**), and boreal fall (SON; panels **m-o**). Vertical error bars in panels **a-c** represent 90% confidence intervals.

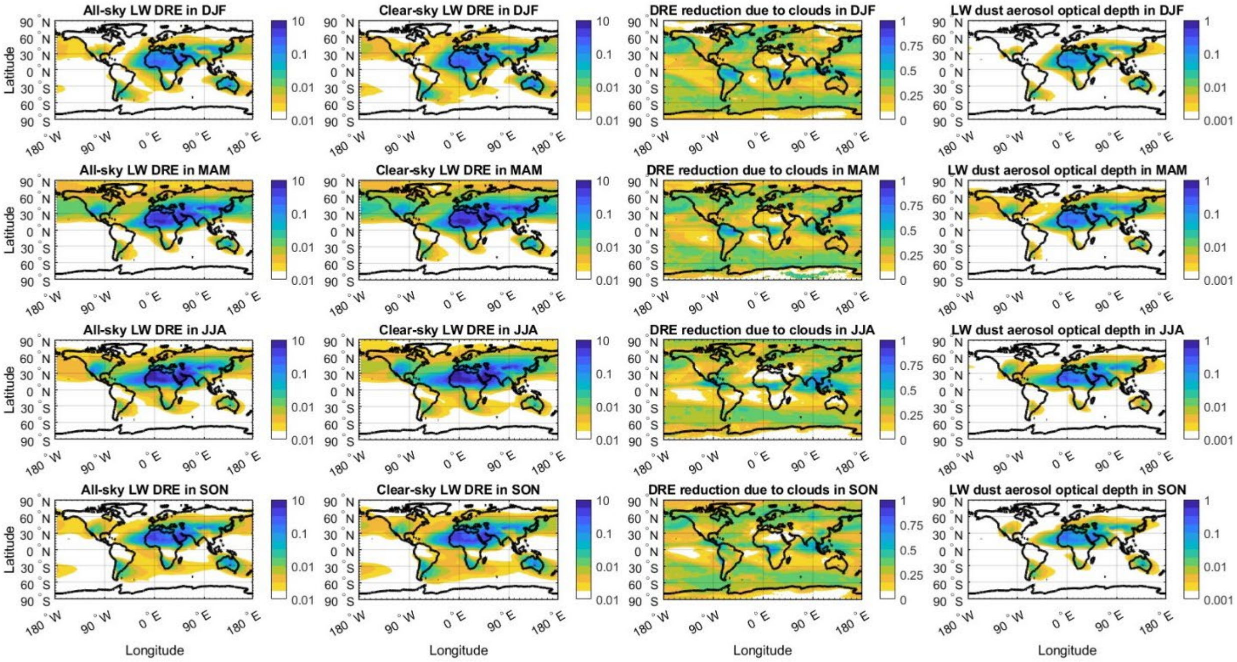

Supplementary Figure 11. Seasonally averaged dust radiative effects in the longwave (LW) spectrum. Shown for all four seasons (different rows) are the all-sky (first column) and clear-sky (second column) LW direct radiative effect (DRE) at the top-of-atmosphere, the fractional reduction of the LW DRE due to cloud cover (third column), and the LW dust aerosol optical depth (fourth column).

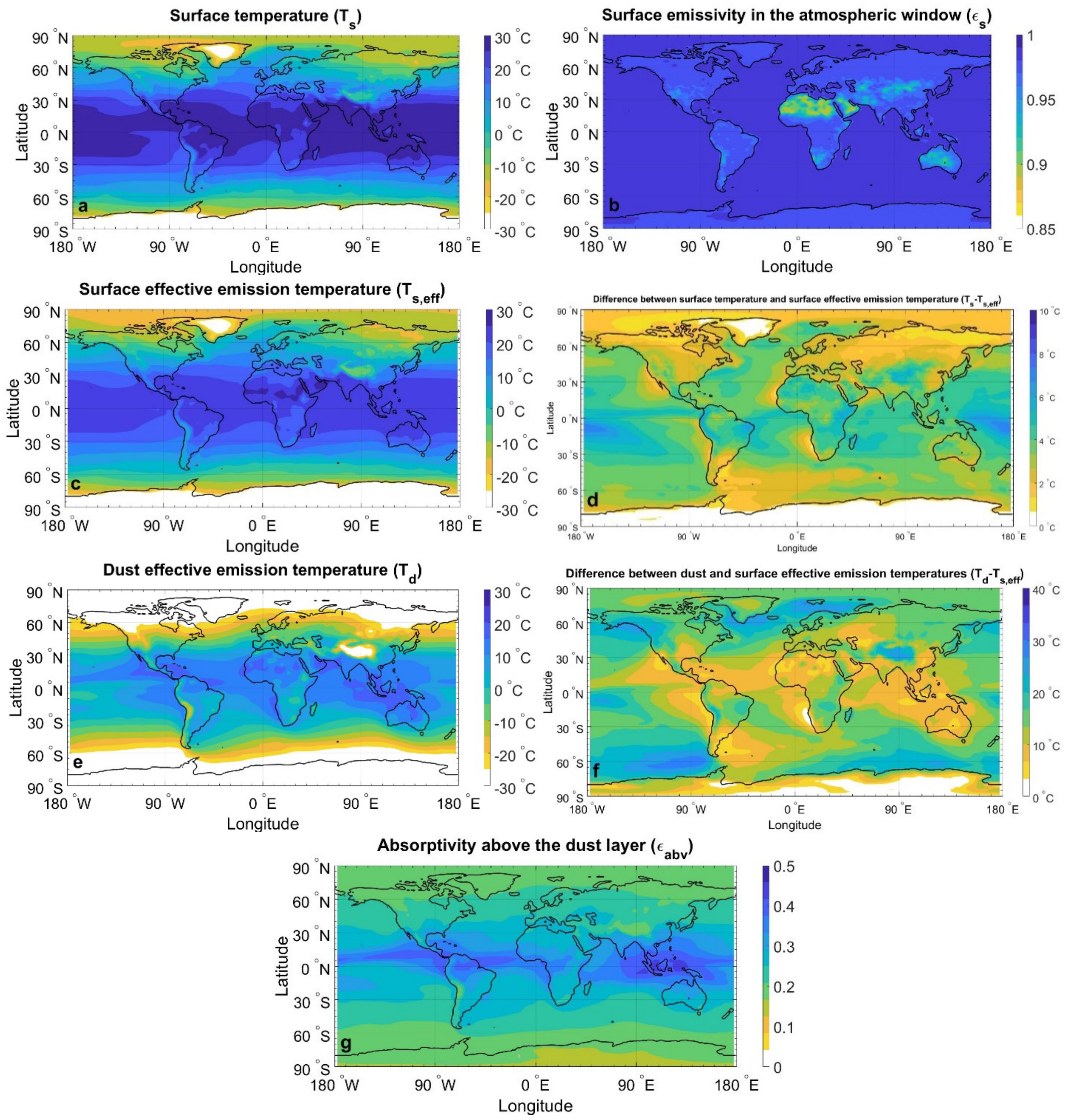

Supplementary Figure 12. Dust radiative effects in the LW spectrum are controlled by temperature, surface emissivity, and atmospheric absorptivity. Shown are the annual mean surface temperature, T_s (**a**), the surface emissivity in the atmospheric window (8-14 μm), $\bar{\epsilon}_s$ (**b**), the effective emission temperature below the dust layer, $T_{s,eff}$ (**c**), the difference between the surface temperature and the surface effective emission temperature, T_s-$T_{s,eff}$ (**d**), the dust effective emission temperature, T_d (**e**), the difference between the dust and the surface effective emission temperature, T_d-$T_{s,eff}$ (**f**), and the absorptivity in the atmospheric window above the dust layer, $\bar{\epsilon}_{abv}$ (**g**). All graphs represent annual mean values for the 2004-2008 period (see Methods).

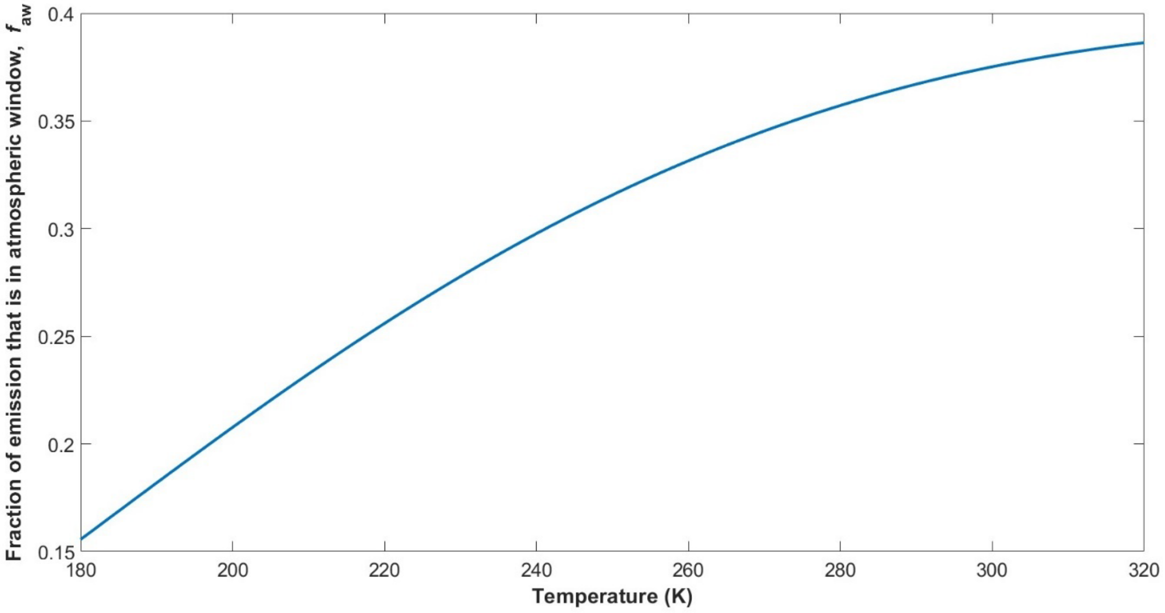

Supplementary Figure 13. Fraction of emitted radiant energy that is in the atmospheric window between 8 and 14 μm, as a function of temperature.

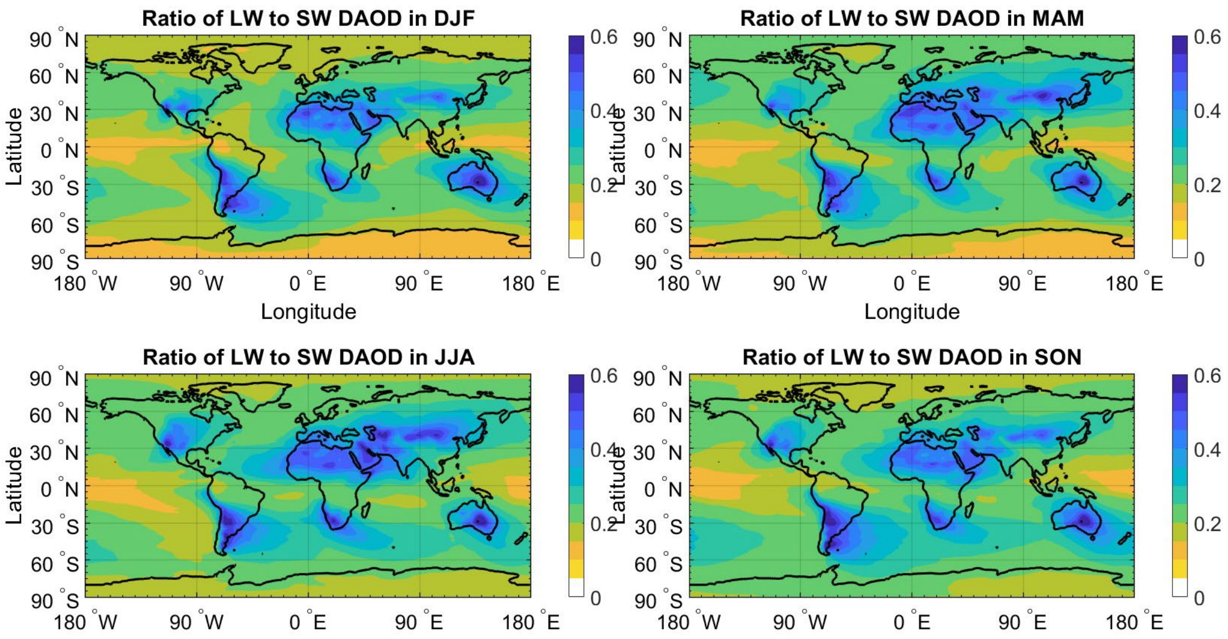

Supplementary Figure 14. Ratio of the longwave (LW) dust aerosol optical depth (DAOD; averaged across the 8-14 μm atmospheric window) and the shortwave (SW) DAOD (at 550 nm) as a function of season.

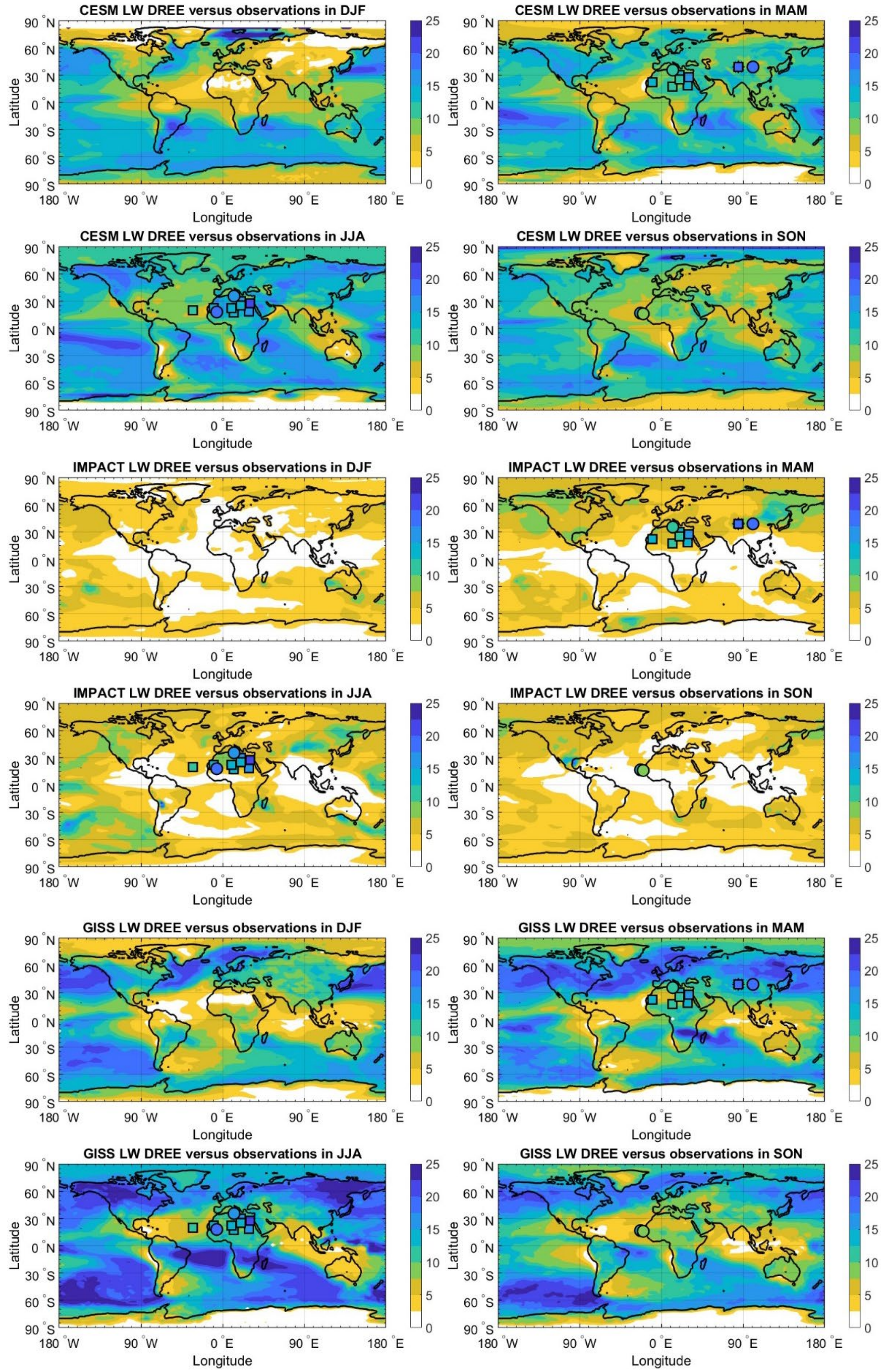
CESM LW DREE versus observations in DJF
CESM LW DREE versus observations in MAM
CESM LW DREE versus observations in JJA
CESM LW DREE versus observations in SON
IMPACT LW DREE versus observations in DJF
IMPACT LW DREE versus observations in MAM
IMPACT LW DREE versus observations in JJA
IMPACT LW DREE versus observations in SON
GISS LW DREE versus observations in DJF
GISS LW DREE versus observations in MAM
GISS LW DREE versus observations in JJA
GISS LW DREE versus observations in SON
Latitude
Longitude
90 °N
60 °N
30 °N
0 °N
30 °S
60 °S
90 °S
180 °W
90 °W
0 °E
90 °E
180 °E
0
5
10
15
20
25

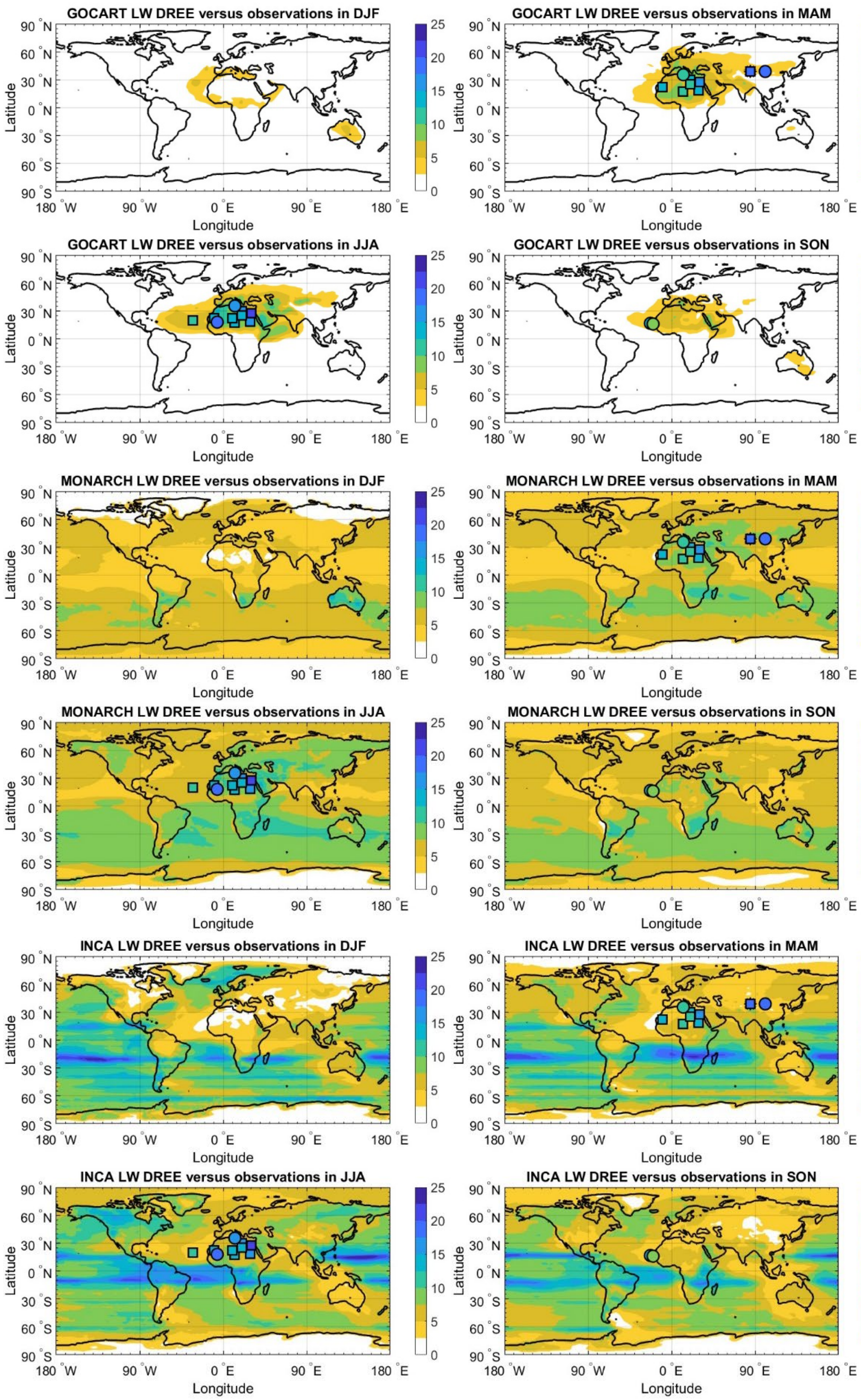
GOCART LW DREE versus observations in DJF
GOCART LW DREE versus observations in MAM
GOCART LW DREE versus observations in JJA
GOCART LW DREE versus observations in SON
MONARCH LW DREE versus observations in DJF
MONARCH LW DREE versus observations in MAM
MONARCH LW DREE versus observations in JJA
MONARCH LW DREE versus observations in SON
INCA LW DREE versus observations in DJF
INCA LW DREE versus observations in MAM
INCA LW DREE versus observations in JJA
INCA LW DREE versus observations in SON
Latitude
Longitude
90 °N
60 °N
30 °N
0 °N
30 °S
60 °S
90 °S
180 °W
90 °W
0 °E
90 °E
180 °E
0
5
10
15
20
25

Supplementary Figure 15. Maps of simulated seasonal longwave (LW) clear-sky direct radiative effect efficiency (DREE) at top-of-atmosphere (TOA) for each of the six models in our ensemble. Colored symbols denote observational estimates of LW clear-sky DREE at TOA derived mainly from in situ (circles) and satellite (squares) data.

Supplementary References

1 Adebiyi, A. A. & Kok, J. F. Climate models miss most of the coarse dust in the atmosphere. *Science Advances* **6**, eaaz9507, doi:10.1126/sciadv.aaz9507 (2020).

2 Adebiyi, A. A. *et al.* Dust Constraints from joint Observational-Modelling-experiMental analysis (DustCOMM): comparison with measurements and model simulations. *Atmospheric Chemistry and Physics* **20**, 829-863, doi:10.5194/acp-20-829-2020 (2020).

3 Kok, J. F. *et al.* Improved representation of the global dust cycle using observational constraints on dust properties and abundance. *Atmospheric Chemistry and Physics* **21**, 8127-8167, doi:10.5194/acp-21-8127-2021 (2021).

4 Kok, J. F. *et al.* Contribution of the world's main dust source regions to the global cycle of desert dust. *Atmospheric Chemistry and Physics* **21**, 8169-8193, doi:10.5194/acp-21-8169-2021 (2021).

5 Kok, J. F. *et al.* Smaller desert dust cooling effect estimated from analysis of dust size and abundance. *Nature Geoscience* **10**, 274-278, doi:10.1038/ngeo2912 (2017).

6 Ridley, D. A., Heald, C. L., Kok, J. F. & Zhao, C. An observationally-constrained estimate of global dust aerosol optical depth. *Atmos. Chem. Phys.* **16**, 15097-15117, doi:10.5194/acp-2016-385 (2016).

7 Wilks, D. S. *Statistical Methods in the Amtospheric Sciences*. 3rd edn, (Academic Press, 2011).

8 Kim, D. *et al.* Sources, sinks, and transatlantic transport of North African dust aerosol: A multimodel analysis and comparison with remote sensing data. *Journal of Geophysical Research-Atmospheres* **119**, 6259-6277, doi:10.1002/2013jd021099 (2014).

9 Gelaro, R. *et al.* The Modern-Era Retrospective Analysis for Research and Applications, Version 2 (MERRA-2). *Journal of Climate* **30**, 5419-5454, doi:10.1175/jcli-d-16-0758.1 (2017).

10 Meng, J. *et al.* Improved Parameterization for the Size Distribution of Emitted Dust Aerosols Reduces Model Underestimation of Super Coarse Dust. *Geophysical Research Letters* **49**, doi:10.1029/2021gl097287 (2022).

11 Adebiyi, A. *et al.* A review of coarse mineral dust in the Earth system. *Aeolian Research* **60**, doi:10.1016/j.aeolia.2022.100849 (2023).

12 Di Biagio, C. *et al.* Global scale variability of the mineral dust long-wave refractive index: a new dataset of in situ measurements for climate modeling and remote sensing. *Atmospheric Chemistry and Physics* **17**, 1901-1929, doi:10.5194/acp-17-1901-2017 (2017).

13 Volz, F. E. Infrared refractive-index of atmospheric aerosol substances. *Applied Optics* **11**, 755-&, doi:10.1364/ao.11.000755 (1972).

14 Volz, F. E. Infrared optical-constants of ammonium sulfate, sahara dust, volcanic pumice, and flyash. *Applied Optics* **12**, 564-568, doi:10.1364/ao.12.000564 (1973).

15 Fouquart, Y. *et al.* Observations of saharan aerosols - results of eclats field experiment .2. Broad-band radiative characteristics of the aerosols and vertical radiative flux divergence. *Journal of climate and applied meteorology* **26**, 38-52 (1987).

16 Hess, M., Koepke, P. & Schult, I. Optical properties of aerosols and clouds: The software package OPAC. *Bulletin of the American Meteorological Society* **79**, 831-844, doi:10.1175/1520-0477(1998)079<0831:opoaac>2.0.co;2 (1998).

17 Di Biagio, C. *et al.* Variability of the infrared complex refractive index of African mineral dust: experimental estimation and implications for radiative transfer and satellite remote sensing. *Atmospheric Chemistry and Physics* **14**, 11093-11116, doi:10.5194/acp-14-11093-2014 (2014).

18 Fratini, G., Ciccioli, P., Febo, A., Forgione, A. & Valentini, R. Size-segregated fluxes of mineral dust from a desert area of northern China by eddy covariance. *Atmos. Chem. Phys.* **7**, 2839-2854 (2007).

19 Reid, J. S. *et al.* Dynamics of southwest Asian dust particle size characteristics with implications for global dust research. *Journal of Geophysical Research-Atmospheres* **113**, D14212, doi:10.1029/2007jd009752 (2008).
20 Kaaden, N. *et al.* State of mixing, shape factor, number size distribution, and hygroscopic growth of the Saharan anthropogenic and mineral dust aerosol at Tinfou, Morocco. *Tellus Ser. B-Chem. Phys. Meteorol.* **61**, 51-63, doi:10.1111/j.1600-0889.2008.00388.x (2009).
21 Sow, M., Alfaro, S. C., Rajot, J. L. & Marticorena, B. Size resolved dust emission fluxes measured in Niger during 3 dust storms of the AMMA experiment. *Atmos. Chem. Phys.* **9**, 3881-3891 (2009).
22 Huang, Y., Kok, J. F., Saito, M. & Munoz, O. Single-scattering properties of ellipsoidal dust aerosols constrained by measured dust shape distributions. *Atmospheric Chemistry and Physics* **23**, 2557-2577, doi:10.5194/acp-23-2557-2023 (2023).
23 Mayer, B. & Kylling, A. Technical note: The libRadtran software package for radiative transfer calculations - description and examples of use. *Atmospheric Chemistry and Physics* **5**, 1855-1877, doi:10.5194/acp-5-1855-2005 (2005).
24 Emde, C. *et al.* The libRadtran software package for radiative transfer calculations (version 2.0.1). *Geoscientific Model Development* **9**, 1647-1672, doi:10.5194/gmd-9-1647-2016 (2016).
25 Anderson, G., Clough, S., Kneizys, F., Chetwynd, J. & Shettle, E. AFGL atmospheric constituent profiles (0-120 km). (Air Force Geophys. Lab., Hanscom Air Force Base, Bedford, Mass., 1986).
26 Stamnes, K., Tsay, S.-C., Wiscombe, W. & Laszlo, I. DISORT, general-purpose Fortran program for discrete-ordinate-method radiative transfer in scattering and emitting layered media: documentation of methodology. (2000).
27 Gasteiger, J. *et al.* Representative wavelengths absorption parameterization applied to satellite channels and spectral bands. *Journal of Quantitative Spectroscopy & Radiative Transfer* **148**, 99-115, doi:10.1016/j.jqsrt.2014.06.024 (2014).
28 Efron, B. *The jackknife, the bootstrap, and other resampling plans*. (Capital City Press, 1982).
29 Chernick, M. R. *Bootstrap methods : a guide for practitioners and researchers*. 400 (Wiley-Interscience, 2007).
30 Neale, R. B. *et al.* Description of the NCAR Community Atmosphere Model (CAM 5.0). *NCAR Technical Note* **TN-486**, pp. 268 (2010).
31 Mahowald, N. M. *et al.* Change in atmospheric mineral aerosols in response to climate: Last glacial period, preindustrial, modern, and doubled carbon dioxide climates. *J. Geophys. Res.* **111**, D10202, doi:10.1029/2005jd006653 (2006).
32 Kok, J. F. A scaling theory for the size distribution of emitted dust aerosols suggests climate models underestimate the size of the global dust cycle. *Proc. Natl. Acad. Sci. U. S. A.* **108**, 1016-1021, doi:10.1073/pnas.1014798108 (2011).
33 Ramanathan, V. & Downey, P. A nonisothermal emissivity and absorptivity formulation for water-vapor. *Journal Of Geophysical Research-Atmospheres* **91**, 8649-8666 (1986).
34 Collins, W., Hackney, J. & Edwards, D. An updated parameterization for infrared emission and absorption by water vapor in the National Center for Atmospheric Research Community Atmosphere Model. *Journal of Geophysical Research-Atmospheres* **107**, doi:10.1029/2001JD001365 (2002).
35 Yoshioka, M. *et al.* Impact of desert dust radiative forcing on Sahel precipitation: Relative importance of dust compared to sea surface temperature variations, vegetation changes, and greenhouse gas warming. *Journal of Climate* **20**, 1445-1467, doi:10.1175/jcli4056.1 (2007).
36 Dufresne, J. L., Gautier, C., Ricchiazzi, P. & Fouquart, Y. Longwave scattering effects of mineral aerosols. *J. Atmos. Sci.* **59**, 1959-1966, doi:10.1175/1520-0469(2002)059<1959:lseoma>2.0.co;2 (2002).

37 Slingo, J. The development and verification of a cloud prediction scheme for the ECMWF model. *Quarterly Journal Of The Royal Meteorological Society* **113**, 899-927 (1987).
38 Klein, S. & Hartmann, D. The seasonal cycle of low stratiform clouds. *Journal of Climate* **6**, 1587-1606 (1993).
39 Xu, K. & Krueger, S. Evaluation of cloudiness parameterizations using a cumulus ensemble model. *Monthly Weather Review* **119**, 342-367 (1991).
40 Vavrus, S. & Waliser, D. An Improved Parametrization for Simulating Arctic Cloud Amount in the CCSM3 Climate Model. *Journal of Climate* **21**, 5673-5687, doi:10.1175/2008JCLI2299.1 (2008).
41 Ito, A., Ye, Y., Yamamoto, A., Watanabe, M. & Aita, M. N. Responses of ocean biogeochemistry to atmospheric supply of lithogenic and pyrogenic iron-containing aerosols. *Geological Magazine* **157**, 741-756, doi:10.1017/s0016756819001080 (2020).
42 Ito, A., Lin, G. X. & Penner, J. E. Radiative forcing by light-absorbing aerosols of pyrogenetic iron oxides. *Scientific Reports* **8**, doi:10.1038/s41598-018-25756-3 (2018).
43 Collins, W. D. *et al.* The Community Climate System Model version 3 (CCSM3). *Journal of Climate* **19**, 2122-2143 (2006).
44 Albani, S. *et al.* Improved dust representation in the Community Atmosphere Model. *J. Adv. Model. Earth Sy.* **6**, 541-570, doi:10.1002/2013ms000279 (2014).
45 Xu, L. & Penner, J. E. Global simulations of nitrate and ammonium aerosols and their radiative effects. *Atmospheric Chemistry and Physics* **12**, 9479-9504, doi:10.5194/acp-12-9479-2012 (2012).
46 Ito, A., Adebiyi, A. A., Huang, Y. & Kok, J. F. Less atmospheric radiative heating by dust due to the synergy of coarser size and aspherical shape. *Atmospheric Chemistry and Physics* **21**, 16869-16891, doi:10.5194/acp-21-16869-2021 (2021).
47 Bauer, S. E. *et al.* Historical (1850-2014) Aerosol Evolution and Role on Climate Forcing Using the GISS ModelE2.1 Contribution to CMIP6. *J. Adv. Model. Earth Syst.* **12**, doi:10.1029/2019ms001978 (2020).
48 Kelley, M. *et al.* GISS-E2.1: Configurations and climatology. *J. Adv. Model. Earth Syst.* **12**, e2019MS002025, doi:10.1029/2019MS002025 (2020).
49 Miller, R. L. *et al.* Mineral dust aerosols in the NASA Goddard Institute for Space Sciences ModelE atmospheric general circulation model. *J. Geophys. Res.-Atmos.* **111**, D06208, doi:10.1029/2005jd005796 (2006).
50 Perlwitz, J. P., Perez Garcia-Pando, C. & Miller, R. L. Predicting the mineral composition of dust aerosols - Part 1: Representing key processes. *Atmos. Chem. Phys.* **15**, 11593-11627 (2015).
51 Ginoux, P. *et al.* Sources and distributions of dust aerosols simulated with the GOCART model. *J. Geophys. Res.* **106**, 20255-20273 (2001).
52 Cakmur, R. V. *et al.* Constraining the magnitude of the global dust cycle by minimizing the difference between a model and observations. *Journal of Geophysical Research-Atmospheres* **111**, D06207, doi:10.1029/2005jd005791 (2006).
53 Cakmur, R. V., Miller, R. L. & Torres, O. Incorporating the effect of small-scale circulations upon dust emission in an atmospheric general circulation model. *Journal of Geophysical Research-Atmospheres* **109**, doi:10.1029/2003jd004067 (2004).
54 Bauer, S. E. & Koch, D. Impact of heterogeneous sulfate formation at mineral dust surfaces on aerosol loads and radiative forcing in the Goddard Institute for Space Studies general circulation model. *Journal of Geophysical Research-Atmospheres* **110**, doi:10.1029/2005jd005870 (2005).
55 Sinyuk, A., Torres, O. & Dubovik, O. Combined use of satellite and surface observations to infer the imaginary part of refractive index of Saharan dust. *Geophysical Research Letters* **30**, 1081, doi:10.1029/2002gl016189 (2003).

56 Patterson, E. M., Gillette, D. A. & Stockton, B. H. Complex index of refraction between 300 and 700 nm for saharan aerosols. *Journal of Geophysical Research-Oceans and Atmospheres* **82**, 3153-3160, doi:10.1029/JC082i021p03153 (1977).

57 Schmidt, G. A. *et al.* Present-day atmospheric simulations using GISS ModelE: Comparison to in situ, satellite, and reanalysis data. *Journal of Climate* **19**, 153-192, doi:10.1175/jcli3612.1 (2006).

58 Twomey, S. Influence of pollution on shortwave albedo of clouds. *J. Atmos. Sci.* **34**, 1149-1152 (1977).

59 Menon, S. & Rotstayn, L. The radiative influence of aerosol effects on liquid-phase cumulus and stratiform clouds based on sensitivity studies with two climate models. *Clim. Dyn.* **27**, 345-356, doi:10.1007/s00382-006-0139-3 (2006).

60 Miller, R. *et al.* CMIP6 Historical Simulations (1850-2014) With GISS-E2.1. *J. Adv. Model. Earth Syst.* **13**, doi:10.1029/2019MS002034 (2021).

61 Forster, P. *et al.* (eds V. Masson-Delmotte *et al.*) Ch. Chapter 7: The Earth's energy budget, climate feedbacks, and climate sensitivity, (2021).

62 DelGenio, A., Yao, M., Kovari, W. & Lo, K. A prognostic cloud water parameterization for global climate models. *Journal of Climate* **9**, 270-304 (1996).

63 DelGenio, A. D. & Yao, M.-S. in *The Representation of Cumulus Convection in Numerical Models AMS Meteorological Monograph* (eds K.A. Emanuel & D.A. Raymond) (American Meteorological Society, 1993).

64 Schmidt, G. *et al.* Configuration and assessment of the GISS ModelE2 contributions to the CMIP5 archive. *J. Adv. Model. Earth Syst.* **6**, 141-184, doi:10.1002/2013MS000265 (2014).

65 Hansen, J. *et al.* Efficient 3-dimensional global-models for climate studies - model-I and model-II. *Monthly Weather Review* **111**, 609-662, doi:10.1175/1520-0493(1983)111<0609:ETDGMF>2.0.CO;2 (1983).

66 Chin, M. *et al.* Tropospheric aerosol optical thickness from the GOCART model and comparisons with satellite and Sun photometer measurements. *J. Atmos. Sci.* **59**, 461-483, doi:10.1175/1520-0469(2002)059<0461:taotft>2.0.co;2 (2002).

67 Colarco, P., da Silva, A., Chin, M. & Diehl, T. Online simulations of global aerosol distributions in the NASA GEOS-4 model and comparisons to satellite and ground-based aerosol optical depth. *Journal of Geophysical Research-Atmospheres* **115**, doi:10.1029/2009jd012820 (2010).

68 Kok, J. F. Does the size distribution of mineral dust aerosols depend on the wind speed at emission? *Atmos. Chem. Phys.* **11**, 10149-10156, doi:10.5194/acp-11-10149-2011 (2011).

69 Colarco, P. R. *et al.* Impact of radiatively interactive dust aerosols in the NASA GEOS-5 climate model: Sensitivity to dust particle shape and refractive index. *Journal of Geophysical Research-Atmospheres* **119**, 753-786, doi:10.1002/2013jd020046 (2014).

70 Koepke, P., Hess, M., Schult, I. & Shettle, E. Global Aerosol Data Set. (Max Planck Institute for Meteorology, Hamburg, 1997).

71 Iacono, M. J. *et al.* Radiative forcing by long-lived greenhouse gases: Calculations with the AER radiative transfer models. *Journal of Geophysical Research-Atmospheres* **113**, doi:10.1029/2008jd009944 (2008).

72 Bacmeister, J., Suarez, M. & Robertson, F. Rain reevaporation, boundary layer-convection interactions, and Pacific rainfall patterns in an AGCM. *J. Atmos. Sci.* **63**, 3383-3403 (2006).

73 Molod, A. Constraints on the Profiles of Total Water PDF in AGCMs from AIRS and a High-Resolution Model. *JOURNAL OF CLIMATE* **25**, 8341-8352, doi:10.1175/JCLI-D-11-00412.1 (2012).

74 Molod, A., Takacs, L., Suarez, M. & Bacmeister, J. Development of the GEOS-5 atmospheric general circulation model: evolution from MERRA to MERRA2. *GEOSCIENTIFIC MODEL DEVELOPMENT* **8**, 1339-1356, doi:10.5194/gmd-8-1339-2015 (2015).

75 Pérez, C. *et al.* Atmospheric dust modeling from meso to global scales with the online NMMB/BSC-Dust model - Part 1: Model description, annual simulations and evaluation. *Atmospheric Chemistry and Physics* **11**, 13001-13027, doi:10.5194/acp-11-13001-2011 (2011).
76 Badia, A. *et al.* Description and evaluation of the Multiscale Online Nonhydrostatic AtmospheRe CHemistry model (NMMB-MONARCH) version 1.0: gas-phase chemistry at global scale. *Geoscientific Model Development* **10**, 609-638, doi:10.5194/gmd-10-609-2017 (2017).
77 Klose, M. *et al.* Mineral dust cycle in the Multiscale Online Nonhydrostatic AtmospheRe CHemistry model (MONARCH) Version 2.0. *Geoscientific Model Development* **14**, 6403-6444, doi:10.5194/gmd-14-6403-2021 (2021).
78 Janjic, Z. & Gall, R. Scientific documentation of the NCEP Nonhydrostatic Multiscale Model on the B grid (NMMB). (National Center for Atmospheric Research, Camp Springs, MD, USA., 2012).
79 Hu, Y. & Stamnes, K. An accurate parameterization of the radiative properties of water clouds suitable for use in climate models. *Journal of Climate* **6**, 728-742 (1993).
80 Fu, Q., Yang, P. & Sun, W. An accurate parameterization of the infrared radiative properties of cirrus clouds for climate models. *Journal of Climate* **11**, 2223-2237 (1998).
81 Di Biagio, C., Balkanski, Y., Albani, S., Boucher, O. & Formenti, P. Direct Radiative Effect by Mineral Dust Aerosols Constrained by New Microphysical and Spectral Optical Data. *Geophysical Research Letters* **47**, doi:10.1029/2019gl086186 (2020).
82 Checa-Garcia, R. *et al.* Evaluation of natural aerosols in CRESCENDO Earth system models (ESMs): mineral dust. *Atmospheric Chemistry and Physics* **21**, 10295-10335, doi:10.5194/acp-21-10295-2021 (2021).
83 Ryder, C. L. *et al.* Optical properties of Saharan dust aerosol and contribution from the coarse mode as measured during the Fennec 2011 aircraft campaign. *Atmospheric Chemistry and Physics* **13**, 303-325, doi:10.5194/acp-13-303-2013 (2013).
84 Morcrette, J.-J., Clough, S. A., Mlawer, E. J. & Iacono, M. J. Impact of a validated radiative transfer scheme, RRTM, on the ECMWF model climate and 10-day forecasts. (ECMWF, Shinfield Park, Reading, 1998).
85 Boucher, O. *et al.* Presentation and Evaluation of the IPSL-CM6A-LR Climate Model. *J. Adv. Model. Earth Syst.* **12**, doi:10.1029/2019ms002010 (2020).
86 Hourdin, F. *et al.* LMDZ6A: The Atmospheric Component of the IPSL Climate Model With Improved and Better Tuned Physics. *J. Adv. Model. Earth Syst.* **12**, doi:10.1029/2019MS001892 (2020).
87 Bony, S. & Emanuel, K. A parameterization of the cloudiness associated with cumulus convection; Evaluation using TOGA COARE data. *J. Atmos. Sci.* **58**, 3158-3183 (2001).
88 Jam, A., Hourdin, F., Rio, C. & Couvreux, F. Resolved Versus Parametrized Boundary-Layer Plumes. Part III: Derivation of a Statistical Scheme for Cumulus Clouds. *Boundary-Layer Meteorology* **147**, 421-441, doi:10.1007/s10546-012-9789-3 (2013).
89 Brindley, H. E. & Russell, J. E. An assessment of Saharan dust loading and the corresponding cloud-free longwave direct radiative effect from geostationary satellite observations. *Journal of Geophysical Research-Atmospheres* **114**, doi:10.1029/2008jd011635 (2009).
90 Yang, E. S., Gupta, P. & Christopher, S. A. Net radiative effect of dust aerosols from satellite measurements over Sahara. *Geophysical Research Letters* **36**, L18812, doi:10.1029/2009gl039801 (2009).
91 Xia, X. & Zong, X. Shortwave versus longwave direct radiative forcing by Taklimakan dust aerosols. *Geophysical Research Letters* **36**, doi:10.1029/2009GL037237 (2009).
92 Haywood, J. *et al.* Can desert dust explain the outgoing longwave radiation anomaly over the Sahara during July 2003? *Journal of Geophysical Research-Atmospheres* **110**, doi:10.1029/2004JD005232 (2005).

93 Liou, K. N. *An Introduction to Atmospheric Radiation*. Second edn, (Academic Press, 2002).
94 Lee, L. A., Reddington, C. L. & Carslaw, K. S. On the relationship between aerosol model uncertainty and radiative forcing uncertainty. *Proc. Natl. Acad. Sci. U. S. A.* **113**, 5820-5827, doi:10.1073/pnas.1507050113 (2016).
95 Zhang, Z., Song, Q., Zheng, J. & Yu, H. Effects of surface coating on the shortwave and longwave radiative effects of dust aerosol in comparison with external mixing: A theoretical study. *Journal of Quantitative Spectroscopy & Radiative Transfer* **324**, doi:10.1016/j.jqsrt.2024.109060 (2024).
96 Li , W. *et al.* Aqueous-phase secondary organic aerosol formation on mineral dust. *National Science Review*, nwaf221, doi:10.1093/nsr/nwaf221 (2025).
97 Kok, J. F. *et al.* Mineral dust aerosol impacts on global climate and climate change. *Nature Reviews Earth & Environment* **4**, 71–86, doi:10.1038/s43017-022-00379-5 (2023).
98 O'Sullivan, D. *et al.* Models transport Saharan dust too low in the atmosphere: a comparison of the MetUM and CAMS forecasts with observations. *Atmospheric Chemistry and Physics* **20**, 12955-12982, doi:10.5194/acp-20-12955-2020 (2020).
99 Gill, T. E., Zobeck, T. M. & Stout, J. E. Technologies for laboratory generation of dust from geological materials. *Journal of Hazardous Materials* **132**, 1-13, doi:10.1016/j.jhazmat.2005.11.083 (2006).
100 Osborne, S. *et al.* Short-wave and long-wave radiative properties of Saharan dust aerosol. *Quarterly Journal of the Royal Meteorological Society* **137**, 1149-1167, doi:10.1002/qj.771 (2011).
101 Heald, C. L. *et al.* Contrasting the direct radiative effect and direct radiative forcing of aerosols. *Atmos. Chem. Phys.* **14**, 5513-5527, doi:10.5194/acp-14-5513-2014 (2014).
102 Woodage, M. & Woodward, S. UK HiGEM: Impacts of Desert Dust Radiative Forcing in a High-Resolution Atmospheric GCM. *JOURNAL OF CLIMATE* **27**, 5907-5928, doi:10.1175/JCLI-D-13-00556.1 (2014).
103 Scanza, R. *et al.* Modeling dust as component minerals in the Community Atmosphere Model: development of framework and impact on radiative forcing. *Atmos. Chem. Phys.* **15**, 537-561 (2015).
104 Klingmuller, K., Lelieveld, J., Karydis, V. A. & Stenchikov, G. L. Direct radiative effect of dust-pollution interactions. *Atmospheric Chemistry and Physics* **19**, 7397-7408, doi:10.5194/acp-19-7397-2019 (2019).
105 Tuccella, P., Curci, G., Pitari, G., Lee, S. & Jo, D. S. Direct Radiative Effect of Absorbing Aerosols: Sensitivity to Mixing State, Brown Carbon, and Soil Dust Refractive Index and Shape. *Journal of Geophysical Research-Atmospheres* **125**, doi:10.1029/2019jd030967 (2020).
106 Li, L. L. *et al.* Quantifying the range of the dust direct radiative effect due to source mineralogy uncertainty. *Atmospheric Chemistry and Physics* **21**, 3973-4005, doi:10.5194/acp-21-3973-2021 (2021).
107 Woodward, S. *et al.* The simulation of mineral dust in the United Kingdom Earth System Model UKESM1. *Atmospheric Chemistry and Physics* **22**, 14503-14528, doi:10.5194/acp-22-14503-2022 (2022).
108 Feng, Y. *et al.* Global Dust Cycle and Direct Radiative Effect in E3SM Version 1: Impact of Increasing Model Resolution. *J. Adv. Model. Earth Syst.* **14**, doi:10.1029/2021MS002909 (2022).
109 Ke, Z., Liu, X., Wu, M., Shan, Y. & Shi, Y. Improved Dust Representation and Impacts on Dust Transport and Radiative Effect in CAM5. *J. Adv. Model. Earth Syst.* **14**, doi:10.1029/2021MS002845 (2022).
110 Wang, H. *et al.* Larger Dust Cooling Effect Estimated From Regionally Dependent Refractive Indices. *Geophysical Research Letters* **51**, doi:10.1029/2023GL107647 (2024).

111 Haywood, J. *et al.* Radiative properties and direct radiative effect of Saharan dust measured by the C-130 aircraft during SHADE: 1. Solar spectrum. *Journal of Geophysical Research-Atmospheres* **108**, doi:10.1029/2002jd002687 (2003).
112 Hansell, R. *et al.* An Assessment of the Surface Longwave Direct Radiative Effect of Airborne Saharan Dust during the NAMMA Field Campaign. *J. Atmos. Sci.* **67**, 1048-1065, doi:10.1175/2009JAS3257.1 (2010).
113 Hansell, R. *et al.* An assessment of the surface longwave direct radiative effect of airborne dust in Zhangye, China, during the Asian Monsoon Years field experiment (2008). *Journal of Geophysical Research-Atmospheres* **117**, doi:10.1029/2011JD017370 (2012).
114 di Sarra, A. *et al.* Shortwave and longwave radiative effects of the intense Saharan dust event of 25-26 March 2010 at Lampedusa (Mediterranean Sea). *Journal of Geophysical Research-Atmospheres* **116**, doi:10.1029/2011JD016238 (2011).
115 Meloni, D. *et al.* Altitude-resolved shortwave and longwave radiative effects of desert dust in the Mediterranean during the GAMARF campaign: Indications of a net daily cooling in the dust layer. *Journal of Geophysical Research-Atmospheres* **120**, 3386-3407, doi:10.1002/2014JD022312 (2015).
116 Meloni, D. *et al.* Determining the infrared radiative effects of Saharan dust: a radiative transfer modelling study based on vertically resolved measurements at Lampedusa. *Atmospheric Chemistry and Physics* **18**, 4377-4401, doi:10.5194/acp-18-4377-2018 (2018).
117 Song, Q. Q. *et al.* Net radiative effects of dust in the tropical North Atlantic based on integrated satellite observations and in situ measurements. *Atmospheric Chemistry and Physics* **18**, 11303-11322, doi:10.5194/acp-18-11303-2018 (2018).
